# Personalized Sequential Nephron Blockade for Diuretic Resistance in Acute Decompensated Heart Failure: A Narrative Review for the Cardiorenal Clinician

**DOI:** 10.3390/jpm16090466

**Published:** 2026-09-09

**Authors:** Guido Gembillo, Concetto Sessa, Luca Zanoli, Luca Visconti, Maria Federica Ricca, Lorenzo Lo Cicero, Andrea Corsonello, Salvatore Petrina, Antonino Nicosia, Luca Soraci, Giuseppe Fede, Guglielmo Piccione, Sebastiano Lumera, Walter Morale, Domenico Santoro

**Affiliations:** 1 Unit of Nephrology and Dialysis, Department of Clinical and Experimental Medicine, University of Messina, 98125 Messina, Italy; mariafedericaricca8@gmail.com (M.F.R.); lorenzolocicero95@gmail.com (L.L.C.); domenico.santoro@unime.it (D.S.); 2 Department of Nephrology and Dialysis, Maggiore Nino Baglieri Hospital, 97015 Modica, Italy; walter.morale@asp.rg.it; 3 Department of Clinical and Experimental Medicine, University of Catania, 95123 Catania, Italy; luca.zanoli@unict.it; 4 Department of Nephrology and Dialysis, Ospedali Riuniti Villa Sofia Cervello, University of Palermo, 90146 Palermo, Italy; lucavisconti2003@yahoo.it; 5 PhD Program in Molecular and Clinical Medicine, University of Palermo, 90100 Palermo, Italy; 6 Unit of Geriatric Medicine, Italian National Research Center on Aging (IRCCS INRCA), 87100 Cosenza, Italy; a.corsonello@inrca.it (A.C.); l.soraci@inrca.it (L.S.); 7 Department of Pharmacy and Health and Nutritional Sciences, University of Calabria, 87036 Rende, Italy; 8 Cardiology Unit, Giovanni Paolo II Hospital, 97100 Ragusa, Italy; smpetrina@gmail.com (S.P.); antonino.nicosia@asp.rg.it (A.N.); 9 Division of Cardiology, Maggiore Nino Baglieri Hospital, 97015 Modica, Italy; giuseppe.fede@asp.rg.it (G.F.); guglielmo.piccione@asp.rg.it (G.P.); 10 Coronary Care Unit (CCU), Department of Cardiology, Riccardo Guzzardi Hospital, 97019 Vittoria, Italy; sebastiano.lumera@asp.rg.it

**Keywords:** heart failure, acute decompensated heart failure, cardiorenal syndrome, diuretic resistance, sequential nephron blockade, furosemide, thiazide diuretics, acetazolamide, SGLT2 inhibitors, natriuresis, decongestion, WNK-SPAK-NCC pathway

## Abstract

Heart failure affects more than 64 million people worldwide, and admissions for acute decompensation account for a substantial proportion of cardiovascular healthcare expenditure. Achieving complete decongestion remains the principal objective of inpatient management, yet many patients are discharged with persistent volume overload, a consistent predictor of early rehospitalization and mortality. Loop diuretics have remained the cornerstone of decongestive therapy for more than six decades; however, their effectiveness is frequently limited by diuretic resistance, leaving many patients incompletely decongested despite dose escalation. Diuretic resistance reflects adaptive sodium retention distributed across multiple nephron segments, a process that extends well beyond the loop itself. Distal-tubular-remodeling-enhanced proximal sodium reabsorption, neurohormonal activation, post-diuretic sodium retention, and impaired tubular drug delivery collectively blunt natriuretic responsiveness, explaining why progressive loop–dose escalation often yields diminishing returns. Sequential nephron blockade provides a physiology-based strategy that matches the adjunct agent to the predominant mechanism of sodium retention. We integrate contemporary randomized evidence with renal tubular physiology to propose a mechanism-based approach: acetazolamide for patients with hypochloremic metabolic alkalosis and enhanced proximal sodium reabsorption, thiazide-type diuretics when distal cotransporter escape predominates, vasopressin antagonists for dilutional hyponatremia complicating congestion, and sodium–glucose cotransporter 2 inhibitors as foundational therapy that combines modest acute natriuresis with durable cardiorenal protection. Early assessment of natriuretic response using spot urine sodium further enables individualized treatment escalation. Together, these concepts move sequential nephron blockade beyond empirical combination diuretic therapy toward a mechanism-based strategy for overcoming diuretic resistance. We propose a personalized, phenotype-driven framework in which early urinary sodium response, kidney function, serum chloride, acid–base status, serum sodium, background SGLT2 inhibitor therapy, and venous congestion imaging guide the selection, escalation, and de-escalation of adjunctive therapy, with the goal of more complete and safer decongestion in acute decompensated heart failure. Matching adjunct choice to a patient’s individual biochemical phenotype, rather than applying diuretics in a fixed sequence, exemplifies precision medicine applied to acute decongestive therapy.

## 1. Introduction

Acute congestive decompensation is a frequent presentation in cardiology and recurs throughout the course of chronic cardiorenal disease. This review focuses primarily on acute decompensated heart failure (ADHF) requiring hospitalization, with particular emphasis on patients who have concurrent chronic kidney disease (CKD). In this subgroup, altered pharmacokinetics of diuretic delivery and an increased risk of inadequate natriuretic response present unique challenges. We integrate physiological principles with available randomized and observational evidence, providing recommendations that reflect expert interpretation rather than formal guideline statements. While the principles discussed have broader applicability, the evidence cited and practical guidance offered are specifically grounded in the context of ADHF with CKD. This clinical burden has persisted across two decades of therapeutic advance. Admission rates for acute decompensation have changed little despite the introduction of disease-modifying therapies that have altered survival [1,2,3]. Angiotensin-converting enzyme (ACE) inhibitors, angiotensin receptor–neprilysin inhibitors (ARNIs), beta-blockers, mineralocorticoid receptor antagonists (MRAs) and sodium–glucose cotransporter 2 (SGLT2) inhibitors (SGLT2is) have improved outcomes in stable HF [4]. Once the renin–angiotensin–aldosterone system (RAAS) and sympathetic activation have been sustained long enough for tubular adaptation to take hold, these agents may not fully reverse the renal sodium avidity that drives repeated decompensation [3,5]. Diuretic therapy for ADHF has historically escalated loop dose or added agents empirically regardless of the underlying mechanism of resistance; a precision medicine approach, selecting adjuncts from each patient’s measurable biochemical phenotype rather than a fixed algorithm, offers a way to match treatment to mechanism in this setting.

Since the 1960s, loop diuretics have been the cornerstone of pharmacological management for acute decongestion. Intravenous administration produces a rapid and potent natriuretic effect, with a dose–response relationship that can be titrated based on urine output. However, early clinical trials did not recognize that chronic inhibition of the sodium-potassium-two-chloride cotransporter (NKCC2) leads to remodeling of the distal nephron, a phenomenon that has only been elucidated in the past decade. These structural changes progressively reduce the net sodium loss achieved with each administered dose [3,5]. As a result, doses that once produced brisk diuresis may eventually yield minimal urine output, leaving patients incompletely decongested at discharge. The concept of sequential nephron blockade, adding agents that target tubular segments upstream or downstream of the loop and disrupting compensatory reabsorption that limits net sodium excretion, has been discussed in the literature for nearly three decades [6]. However, robust randomized evidence supporting its clinical application has emerged only more recently. Recent trial data have begun to clarify which drug combinations can restore natriuresis and what electrolyte and renal trade-offs are involved. Nevertheless, the comparative efficacy of different adjuncts and the optimal matching of patients to specific agents remain uncertain, as adequately powered head-to-head trials are lacking. Over the same period, the molecular framework underlying these combination strategies has also been consolidated.

This narrative review draws on a non-systematic search of PubMed and Embase, supplemented by the reference lists of key articles and by current cardiology and nephrology guidelines. Searches combined terms for heart failure, cardiorenal syndrome, diuretic resistance, and sequential nephron blockade with the names of individual agents and pivotal trials and were restricted to English-language publications, with emphasis on randomized controlled trials, landmark observational studies, and mechanistic investigations relevant to acute decompensated heart failure. The literature search covered publications from database inception to April 2026. Foundational earlier work was retained where it remained physiologically informative, with priority given to recent randomized evidence. The selection reflects expert appraisal rather than a formal systematic protocol, and no quantitative synthesis was undertaken.

## 2. Pharmacology of Diuretics: Sites of Action and Agent-Specific Properties

The transport proteins responsible for sodium reabsorption are distributed unevenly along the nephron, and each segment adapts differently to chronic sodium loading or prolonged pharmacological inhibition. Consequently, two patients receiving identical doses of a loop diuretic may exhibit markedly different natriuresis depending on the dominant site and stage of this tubular adaptation.

### 2.1. Loop Diuretics: Furosemide, Torsemide, Bumetanide, Ethacrynic Acid

The loop diuretics inhibit NKCC2 on the apical membrane of the thick ascending limb of Henle (TALH), which normally manages 20–25% of filtered sodium [5]. The pharmacodynamic consequences extend well beyond the TALH itself. The loss of the medullary concentrating gradient impairs free water reabsorption in the collecting duct, reducing urinary concentration even against the work of a background of antidiuretic hormone (ADH). By eliminating luminal potassium recycling through the renal outer medullary potassium channel (ROMK), loop diuretics abolish the lumen-positive transepithelial potential that normally drives paracellular calcium and magnesium reabsorption, an effect that becomes clinically significant during combination therapy when magnesium depletion renders concurrent hypokalemia refractory to supplementation [5].

Furosemide has considerable pharmacokinetic variability. Oral bioavailability averages 40–50% in ambulatory patients, with wide variability across individuals (range 10–90%) influenced by food intake, bowel wall edema, and gastric motility. In acute decompensation with intestinal edema bioavailability falls further, a pharmacokinetic obstacle that can be avoided by intravenous administration. Protein binding exceeds 95%, primarily to albumin [5]. Furosemide reaches its site of action by active secretion from plasma into the tubular lumen via organic anion transporters 1 and 3 (OAT1, OAT3) on the basolateral membrane of proximal tubular cells. Luminal drug availability depends on this secretory step with plasma concentration contributing only marginally. Plasma half-life ranges from 0.5 to 2 h under normal renal function and lengthens to a mean of approximately 9–10 h in advanced renal failure, with individual values up to about 24 h. Approximately half of the administered dose is excreted unchanged in urine by glomerular filtration and active tubular secretion, the rest metabolized to the acyl glucuronide by UDP-glucuronosyltransferase enzymes UGT1A9 and UGT1A1 in both the kidney and liver [5]. Renal clearance and intrarenal glucuronidation decline in parallel with tubular mass, whereas hepatic glucuronidation is preserved. Plasma drug and its active glucuronide accumulate with a falling glomerular filtration rate (GFR). The drug delivered to the luminal site of action falls because OAT secretory capacity declines with tubular mass and is further compromised by competition from retained uremic organic anions (see Section 3.1). For these reasons, the dose escalation required in CKD reflects defective intraluminal delivery, while intrinsic NKCC2 efficacy remains intact [5].

Torsemide has an oral bioavailability of approximately 80%, largely independent of food or bowel edema, a half-life of 3–4 h under normal renal function, and predominantly hepatic elimination via CYP2C9, making its pharmacokinetics more predictable than those of furosemide, particularly in CKD. Oral-to-intravenous conversion is approximately 1:1 given the high oral bioavailability [5]. The CYP2C9 route introduces interaction risks relevant in cardiorenal polypharmacy. Co-administration with warfarin, which depends on the same isoenzyme for stereoselective metabolism of the active S-enantiomer, has been associated with international normalized ratio (INR) potentiation in case-level reports. However, systematic data show that clinically meaningful interactions are present only in a minority of patients, supporting the idea of INR monitoring rather than empirical dose reduction at initiation [7,8]. Moderate CYP2C9 inhibitors, including amiodarone and fluconazole, can reduce torsemide clearance and raise serum concentrations, with potential for exaggerated natriuresis, volume depletion, and hypotension [8]. Despite the pharmacokinetic advantages, TRANSFORM-HF established clinical equivalence with furosemide on hard outcomes [9,10].

Bumetanide has an oral bioavailability of approximately 80–95%, with more consistent absorption than furosemide, and a plasma half-life of 1 to 1.5 h under normal renal function, with modest prolongation in advanced CKD. Elimination is predominantly renal (about 80% of the dose appears in urine, roughly half as unchanged drug and the remainder as hepatic side-chain oxidation metabolites also excreted renally), while biliary excretion accounts for only about 2% [5]. On a milligram basis, bumetanide is approximately 40 times more potent than furosemide, giving a clinical conversion of furosemide 40 mg ≈ bumetanide 1 mg ≈ torsemide 20 mg, with considerable between-patient variability around these population averages; bumetanide starting doses of 0.5–1 mg are common in elderly or volume-depleted patients [5]. The short half-life argues for twice- or three-times-daily dosing when the goal is sustained luminal exposure above the natriuretic threshold, rather than the once-daily schedule usually adequate for torsemide.

Ethacrynic acid is the only non-sulfonamide loop diuretic in clinical use. Its mechanism is identical (NKCC2 blockade), but its rate of ototoxicity is considered substantially higher than that of furosemide or bumetanide on the basis of historical clinical experience, and severe gastrointestinal intolerance is common. Clinical use is now confined almost entirely to patients with a documented sulfonamide allergy who cannot tolerate furosemide, bumetanide, or torsemide. Elimination is predominantly renal with a smaller biliary component; comparative human pharmacokinetic data is scarce.

### 2.2. Thiazide and Thiazide-like Diuretics: Hydrochlorothiazide, Chlorothiazide, Chlorthalidone, Indapamide, Metolazone

This class inhibits the Na-Cl cotransporter (NCC) in the early distal convoluted tubule (DCT), which handles 5–7% of filtered sodium reabsorption. Intrinsic natriuretic potency is modest. The class matters for diuretic resistance because chronic loop diuretic exposure hypertrophies DCT cells and upregulates NCC, the structural substrate of the braking phenomenon, positioning the DCT precisely where thiazide augmentation exerts its effect [5,6].

Hydrochlorothiazide (HCTZ) has an oral bioavailability of 60–80%, a half-life of 6–15 h, and is excreted predominantly unchanged in urine. The natriuretic effect of NCC inhibition attenuates progressively as eGFR falls, with the CLOROTIC post hoc data quantifying this gradient (Section 4.2). Chlorthalidone retains pharmacodynamic activity in stage 4 CKD on blood pressure endpoints (CLICK [11]). Its diuretic use in ADHF at a low eGFR rests on observational data and pharmacological extrapolation. Metolazone (with a possible additional proximal effect described in older mechanistic work) and chlorthalidone are therefore the pharmacologically preferable DCT-blocking adjuncts at an eGFR < 30; HCTZ 100 mg/day, the dose used at an eGFR < 20 in CLOROTIC, is a defensible alternative when these are unavailable, with intensified electrolyte and renal function monitoring. At doses used for SNB augmentation (25–50 mg/day), HCTZ produces reliable distal sodium blockade in patients with preserved renal function. Chronic HCTZ exposure, particularly during combination therapy or in CKD, is associated with a predictable pattern of manifestation such as hyponatremia, hypokalemia, hypomagnesemia, and (through enhanced passive calcium reabsorption in the proximal tubule) hypercalcemia [12,13]. Close biochemical surveillance during SNB is consistent with the current consensus guidance on natriuresis-guided diuretic titration [14].

Chlorothiazide is the only intravenous thiazide available, reaching peak tubular concentrations within minutes of infusion, which makes pre-dosing before the loop diuretic unnecessary. The standard clinical dose is 500–1000 mg intravenously, administered as a single infusion or in divided doses. Comparative data suggests equivalent diuretic efficacy to oral metolazone [15] but at a substantially higher cost. It is appropriate when oral absorption is severely compromised.

Chlorthalidone differs structurally from the thiazides, binding the NCC at the same site but with characteristics that distinguish it pharmacokinetically. Its oral bioavailability is approximately 65–75% and its half-life is 40–60 h, reflecting a volume of distribution approximately 5–10 times larger than hydrochlorothiazide, principally due to high red blood cell affinity. This prolonged duration makes once-daily dosing sufficient for sustained NCC blockade and provides a pharmacokinetic argument for its use in outpatient combination therapy. Chlorthalidone retains meaningful efficacy at an eGFR as low as 20–30 mL/min/1.73 m^2^, based primarily on CKD hypertension data. However, prospective evidence in acute heart failure (AHF) specifically is limited, giving it broader applicability than HCTZ in the CKD population [5,16].

Indapamide, a sulfonamide-derived thiazide-like compound, has a dual mechanism: NCC inhibition and a direct vasodilatory effect mediated by calcium channel antagonism and prostaglandin synthesis. Oral bioavailability is approximately 93%, peak plasma concentrations occur at approximately 2 h, and the terminal half-life is 14–18 h for the immediate-release formulation (the sustained-release formulation has a longer apparent half-life of approximately 18–24 h). Indapamide is extensively hepatically metabolized to inactive metabolites, with negligible intact drug excreted by the kidney; renal dosage adjustment is therefore not required, and it retains activity in moderate CKD. Clinical data supporting its use specifically in SNB protocols for heart failure are more limited than for metolazone or chlorthalidone.

Metolazone is a quinazoline-derived thiazide-like agent whose profile accounts for its clinical dominance in SNB. Oral bioavailability averages 50–60%; absorption is delayed and unpredictable in gut edema. Elimination half-life varies across primary sources, averaging 8 to 14 h in a biphasic pattern, with a prolonged terminal phase that can exceed 24 h in severe renal impairment and promote accumulation on repeated dosing. The primary site of action is NCC in the early DCT, with a modest additional proximal action documented in older mechanistic work; the proximal component has been proposed to explain continued natriuresis at eGFR values well below 30 mL/min/1.73 m^2^ where other thiazides lose efficacy, although direct contemporary clinical demonstration is limited [5]. Preserved activity at a low GFR, combined with oral availability, underlies metolazone’s wide use as the SNB adjunct in North American practice, while European centers more often rely on hydrochlorothiazide or bendroflumethiazide. The standard starting dose in ADHF is 2.5–5 mg orally, with up to 10 mg in advanced CKD or established diuretic resistance. Pre-dosing before the intravenous loop bolus is pharmacokinetically rational [17]; timing is detailed in Section 6.4. Dosing more frequently than once daily risks cumulative electrolyte depletion given the prolonged terminal half-life.

### 2.3. Mineralocorticoid Receptor Antagonists: Spironolactone, Eplerenone, Finerenone

The three mineralocorticoid receptor antagonists share a pharmacological endpoint. They target the blockade of aldosterone-mediated upregulation of the epithelial sodium channel (ENaC) and the basolateral sodium-potassium ATPase in principal cells of the connecting tubule and cortical collecting duct. However, their differences in structural form produce clinical profiles that are not interchangeable. The collecting duct handles only 2–5% of filtered sodium, so direct natriuretic potency is modest in all three; their importance in heart failure rests primarily on neurohormonal modulation, anti-fibrotic effects and potassium conservation [5].

Spironolactone is a steroidal, non-selective MRA derived from progesterone. It binds the mineralocorticoid receptor (MR) with high affinity but also cross-reacts with androgen and progesterone receptors, producing characteristic side effects such as gynecomastia or breast pain in males (10% of patients in RALES [18]), menstrual irregularities, and loss of libido. The drug is rapidly metabolized to active sulfur-retaining and sulfur-free metabolites (principally canrenone, t½ ~14–24 h, and 7α-thiomethylspirolactone, t½ ~13.8 h [19,20]), which prolong its effective duration; canrenone retains MR-blocking activity at lower per-mole potency than the parent compound, and despite a parent half-life of approximately 1.4 h, the combined parent-plus-metabolite pharmacodynamic effect is sustained for 24–48 h. Oral bioavailability is variable and is roughly doubled by co-administration with food. Spironolactone accumulates preferentially in the kidney relative to the heart [5]. Hyperkalemia risk with a reduced eGFR reflects impaired distal potassium secretion and reduced drug clearance rather than tissue distribution per se [5,21]. RALES established a 30% reduction in all-cause mortality with spironolactone at 25 mg/day (up-titration to 50 mg/day permitted) in severe heart failure with a reduced ejection fraction (HFrEF; left ventricular ejection fraction (LVEF) ≤ 35%) on a background ACE inhibitor and loop diuretic [18].

Canrenone, the pharmacologically active metabolite of spironolactone, is also available as potassium canrenoate and to date is the only MRA licensed for intravenous administration in several European countries [22,23]. Potassium canrenoate is a water-soluble prodrug that undergoes spontaneous in vivo cyclization to canrenone. The plasma elimination half-life of canrenone is approximately 13.5–24 h in patients without heart failure or hepatic dysfunction, lengthening to a mean of 37 h in congestive heart failure and 59 h in hepatic disease; these kinetics support once- or twice-daily intravenous dosing in acute decompensation [19,20]. The European prescribing information for potassium canrenoate specifies an intravenous dose range of 200–600 mg/day (1–3 vials of 200 mg) titrated to response, with a ceiling of 800 mg/day, and lists severe hyperkalemia, severe hyponatremia, and severe renal failure as contraindications. Treatment should be discontinued when serum sodium falls below 126 mmol/L or potassium exceeds 5 mmol/L. Receptor binding is identical to spironolactone, with the same off-target endocrine risk. Potassium canrenoate is the only feasible route of MRA delivery when oral intake is precluded by intestinal edema, impaired consciousness, or vomiting. A retrospective single-center study of 100 patients with ADHF on a standardized intravenous diuretic protocol reported tolerability of potassium canrenoate at mean daily doses of 198 mg (low-dose, range 100–280 mg) and 360 mg (high-dose, range 300–600 mg), with no significant excess of hyperkalemia, worsening renal function, or mortality versus conventional dosing. In this cohort the upper-range doses were administered in a minority of patients rather than as a routine recommendation, and prospective efficacy data are not available [23]. Potassium canrenoate is not approved in the United States.

Eplerenone is a steroidal MRA with greater MR selectivity than spironolactone. It has a lower androgen receptor and progesterone receptor affinity [24], substantially reducing sex-hormone-related adverse effects. Oral bioavailability is approximately 69%, with a half-life 4–6 h, and with extensive CYP3A4 metabolism to inactive metabolites. Strong CYP3A4 inhibitors raise systemic exposure approximately fivefold and are contraindicated, while moderate inhibitors (verapamil, diltiazem, erythromycin, fluconazole) produce a two- to threefold rise and cap the daily dose at 25 mg [24]. Concurrent ACE inhibitors, angiotensin receptor blockers (ARBs), or potassium supplements amplify the hyperkalemia risk, as established in EPHESUS and EMPHASIS-HF. On this basis, serum potassium and creatinine should be monitored before initiation, within the first week, at one month, and at each subsequent dose adjustment [25,26,27]. In vitro MR affinity is roughly an order of magnitude lower for eplerenone than for spironolactone; nevertheless, effective doses in EPHESUS and EMPHASIS-HF (25–50 mg/day) match those of spironolactone in RALES. These results reflect receptor-saturation plateau behavior rather than per-milligram equipotency. EMPHASIS-HF showed a significant reduction in cardiovascular death and HF hospitalization with eplerenone 25–50 mg/day in mildly symptomatic HFrEF (NYHA II), supporting earlier initiation than the severe HF population studied in RALES [28].

Finerenone is a non-steroidal MRA. In vitro, its affinity for the MR is intermediate between eplerenone and spironolactone, with greater MR selectivity than either steroidal agent over androgen, progesterone, glucocorticoid, and thyroid hormone receptors. The pharmacokinetics differ from those of the steroidal MRAs. Oral bioavailability is approximately 43%, with a half-life 2–3 h, no active metabolites, and equal tissue distribution between the heart and kidney rather than the renal predominance of spironolactone [29,30]. Whether the differential distribution translates into a more favorable hyperkalemia tolerability profile is a pharmacological hypothesis rather than an established clinical fact. FINEARTS-HF showed that finerenone raised the hazard of serum potassium > 5.5 mmol/L, approximately twofold relative to placebo [31], with potassium > 6.0 mmol/L in 3.0% versus 1.4%, confirming that hyperkalemia is an MR-antagonism class effect regardless of steroidal versus non-steroidal scaffold [31,32]. The phase IIb dose-finding ARTS-HF trial showed N-terminal pro-B-type natriuretic peptide (NT-proBNP) reduction in a similar proportion of patients with finerenone versus eplerenone, with a numerically lower incidence of hyperkalemia and worsening renal function at the higher finerenone doses and a directional signal toward fewer events in the prespecified exploratory composite of all-cause mortality, cardiovascular hospitalization and emergency presentation for worsening heart failure; the trial was not designed as a formal non-inferiority comparison [33]. FINEARTS-HF showed that finerenone, titrated to 20 or 40 mg once daily by a baseline eGFR, reduces the primary composite of total worsening HF events and cardiovascular death in heart failure with a preserved ejection fraction (HFpEF) and heart failure with a mildly reduced ejection fraction (HFmrEF), with the benefit driven predominantly by fewer worsening HF events and cardiovascular death as an individual endpoint not significantly reduced; this is a population in which the earlier TOPCAT trial of spironolactone was overall neutral, with a post hoc Americas-region signal whose interpretation is still contested [32,34]. Regulatory approvals for HF with an LVEF ≥ 40% (US Food and Drug Administration in July 2025, European Commission in March 2026 following European Medicines Agency (EMA) positive opinion) represent the first MRA approval specifically targeting this phenotype. The finerenone prescribing information does not recommend initiation below an eGFR of 25 mL/min/1.73 m^2^ or with serum potassium > 5.0 mmol/L and contraindicates concomitant strong CYP3A4 inhibitors. Recommended dosing in heart failure with an LVEF ≥ 40% mirrors the FINEARTS-HF protocol: 10 mg once daily titrated to 20 mg with an eGFR 25 to <60 mL/min/1.73 m^2^; 20 mg once daily titrated to 40 mg with an eGFR ≥ 60; and titration at 4-week intervals against potassium and renal function. The CKD with type 2 diabetes mellitus (T2DM) indication shares the 20 mg once-daily target, with a stratified starting dose (10 mg if eGFR 25 to <60 mL/min/1.73 m^2^; 20 mg if eGFR ≥ 60) titrated against potassium and renal function. The short half-life produces a diurnal pattern of MR blockade with washout before the next dose, in contrast with the sustained 24 h coverage afforded by the active metabolites of spironolactone.

In the specific context of SNB and diuretic resistance management, MRAs serve two overlapping roles: direct natriuresis at the collecting duct (modest but additive) and suppression of aldosterone-driven sodium avidity in the distal nephron that compounds loop diuretic resistance. In HFrEF with an eGFR ≥ 30 mL/min/1.73 m^2^ and potassium ≤ 5.0 mmol/L (European Society of Cardiology [ESC] 2021 thresholds [35]), spironolactone 25–50 mg offers the best-established outcomes evidence and lowest cost. In patients with clinically significant gynecomastia or sexual dysfunction on spironolactone, eplerenone at equivalent MR-blocking doses is a reasonable substitute. Where oral intake is precluded during acute decompensation, potassium canrenoate 100–200 mg intravenously once or twice daily provides a comparable steroidal MRA effect in countries where it is licensed, with escalation to 300–400 mg/day in selected refractory cases on the basis of a single observational cohort, under close biochemical surveillance [23]. In patients with concurrent diabetic kidney disease (DKD) and albuminuria, finerenone satisfies both RAAS inhibition and renal protection mandates, with the caveats on comparative tolerability noted above [22,29].

### 2.4. ENaC Blockers: Amiloride and Triamterene

Amiloride and triamterene are the two clinically available direct ENaC blockers. Both occlude the channel pore at the luminal membrane of principal cells in the late DCT and cortical collecting duct, bypassing aldosterone-dependent transcription entirely. In patients with primary or secondary hyperaldosteronism whose aldosterone-driven ENaC activity exceeds what tolerable MRA doses can suppress, direct ENaC blockade adds a meaningful effect [36]. Natriuretic potency is weak, as the collecting duct handles under 5% of filtered sodium, but potassium sparing is marked and acts directly at the channel pore.

Amiloride is a pyrazinoylguanidine derivative with oral bioavailability of approximately 50%, peak plasma concentration at 3–4 h, and a half-life of 6–9 h. It is not metabolized hepatically and is eliminated unchanged via glomerular filtration and tubular secretion, so its half-life prolongs predictably with a declining eGFR; dose reduction is required below an eGFR of 30 mL/min/1.73 m^2^. Amiloride does not cross-react with sex hormone receptors and is free of clinically significant endocrine adverse effects. Outside the heart-failure setting, PATHWAY-3 showed that combining amiloride with hydrochlorothiazide attenuates the thiazide-induced rise in plasma glucose while preserving antihypertensive efficacy [37], a finding relevant to long-term outpatient continuation. Protease-mediated ENaC activation in nephrotic syndrome, in which filtered serine proteases (plasmin, prostasin) cleave and constitutively activate ENaC, is directly inhibited by amiloride; clinical evidence supporting amiloride in nephrotic edema refractory to conventional diuretics is limited to small series and pathophysiological reasoning [5].

Triamterene shares the same mechanism as amiloride but differs in several pharmacologically significant ways. Oral bioavailability is variable (reported range approximately 30–70%) and increases substantially when taken with a high-fat meal. Routine co-administration with hydrochlorothiazide exploits complementary potassium saving. The parent compound has a plasma half-life of 1.5–2.5 h, with the active 4-hydroxytriamterene sulfate metabolite extending the pharmacodynamic effect: onset of action is 2–4 h and duration is 12–16 h, although the diuretic effect may not stabilize for several days of continuous dosing. The active metabolite is renally eliminated; consequently, both hepatic dysfunction and renal dysfunction can cause drug accumulation, and triamterene is generally avoided when the eGFR is substantially reduced. A clinically relevant distinction from amiloride is that amiloride conserves magnesium whereas triamterene increases urinary magnesium excretion, based on pharmacological rationale and limited clinical observation [5]. This makes amiloride pharmacologically preferable when hypomagnesemia is already a concern during SNB with loop and thiazide agents. Triamterene crystalline nephropathy, a cast formation from triamterene crystals in the distal tubule, is a recognized rare complication that can present as nephrolithiasis or acute tubular injury, and concomitant NSAID use has been described as a contributing factor in case reports and small series [38].

### 2.5. Carbonic Anhydrase Inhibitors: Acetazolamide

Acetazolamide inhibits carbonic anhydrase (CA) isoforms II and IV in the proximal tubule. CA-II is cytosolic and catalyzes the interconversion of CO_2_ and carbonic acid intracellularly, while CA-IV is GPI-anchored to the brush border and catalyzes the same reaction in the tubular lumen. Inhibition of both abolishes the coupled mechanism that drives proximal sodium bicarbonate reabsorption. The proximal tubule normally accounts for approximately 65–70% of filtered sodium reabsorption [5]. Neurohormonal activation in heart failure further enhances proximal reabsorption, reducing sodium delivery to the loop and limiting the substrate available for NKCC2 inhibition. Acetazolamide alone produces only a mild natriuresis for this reason: downstream segments recover much of the additionally delivered sodium. Its role in SNB is upstream facilitation: by restoring sodium delivery to the loop and distal nephron, acetazolamide increases the substrate available for inhibition at NKCC2 and at downstream segments, with no change in luminal furosemide concentration [39,40]. In combination with a loop diuretic, this proximal contribution becomes pharmacodynamically meaningful, as a prespecified natriuresis analysis of ADVOR demonstrated, complementing the trial’s primary decongestion endpoint [39,40].

Acetazolamide has an oral bioavailability of approximately 90%, has a half-life of 6–9 h, and is eliminated unchanged by the kidney. The biochemical consequence of carbonic anhydrase inhibition, metabolic acidosis and urinary alkalinization, is pharmacodynamically therapeutic against the hypochloremic metabolic alkalosis that develops during sustained loop and thiazide therapy. The historical labeled indication for edema in congestive heart failure specifies 250–375 mg (5 mg/kg) once daily with intermittent (alternate-day) dosing. The 500 mg/day intravenous regimen used for up to three consecutive days in ADVOR exceeds historical labeled dosing and represents a trial-derived rather than label-directed schedule; the practice recommendation here follows ADVOR’s randomized evidence [39].

Two caveats qualify external validity. First, the pre-admission outpatient loop diuretic dose in ADVOR was a median of approximately 60 mg/day oral furosemide equivalent [39], substantially lower than in patients with established outpatient diuretic tolerance. Second, the ADVOR protocol explicitly excluded patients on an SGLT2i at randomization, among other proximal tubular diuretics, so the contemporary population now treated with SGLT2 inhibition is unrepresented in the trial. Whether acetazolamide adds proximal benefit beyond that already conferred by SGLT2 inhibition, which independently raises sodium chloride delivery to the macula densa, remains an open question. DEA-HF [41] addressed this question indirectly in 42 ambulatory patients with congestion refractory to high-dose oral diuretics, nearly all of whom were receiving an SGLT2i, randomized in an open-label crossover design to intravenous furosemide 250 mg alone, with added oral metolazone 5 mg or with added intravenous acetazolamide 500 mg. Metolazone produced the highest natriuresis, whereas acetazolamide produced numerically lower natriuresis than furosemide alone and significantly less than metolazone. Two non-exclusive explanations for the absent acetazolamide signal were proposed: the high in-trial furosemide dose (250 mg per session, against a median in-trial intravenous furosemide of approximately 120 mg/24 h in ADVOR) potentially masking any proximal increment and convergent action at the proximal tubule with background SGLT2 inhibition, both raising NaCl delivery to the macula densa through distinct transporters [41]. With 42 patients in an open-label crossover and an ambulatory setting, DEA-HF is hypothesis-generating and cannot adjudicate the ADVOR effect size in acute decompensation.

### 2.6. SGLT2 Inhibitors and Sotagliflozin

SGLT2, expressed predominantly in the early proximal tubule, recaptures approximately 90% of filtered glucose alongside sodium. Under euglycemic conditions, the absolute amount of sodium recaptured via this transporter is modest relative to total proximal reabsorption, which is why SGLT2 inhibition produces a smaller direct natriuretic effect per dose than either loop diuretics or thiazides. SGLT2 inhibition increases NaCl delivery to the macula densa; through tubuloglomerular feedback, this augments the adenosine-mediated afferent arteriolar tone, reducing the single-nephron GFR and intraglomerular pressure, an effect that attenuates hyperfiltration-driven glomerular injury and slows CKD progression independently of natriuresis or blood pressure [42,43].

Among SGLT2is, only three agents have heart-failure-specific outcome evidence relevant to sequential nephron blockade: empagliflozin (EMPEROR-Reduced, EMPEROR-Preserved, EMPULSE), dapagliflozin (DAPA-HF, DELIVER, DICTATE-AHF, DAPA-RESIST), and sotagliflozin (SOLOIST-WHF). Canagliflozin and ertugliflozin have not been tested in dedicated heart failure trials of comparable scope and are not discussed further as decongestive adjuncts. Empagliflozin and dapagliflozin are the two agents with the widest evidence base in heart failure across the ejection fraction spectrum. Oral bioavailability is around 78% for dapagliflozin and around 75% for empagliflozin, half-lives lie between 12 and 17 h, and both agents are metabolized primarily via UDP-glucuronosyltransferase-mediated conjugation rather than CYP450, reducing the risk of drug–drug interactions in polypharmacy populations. Glucosuria and the associated osmotic diuresis decline proportionally with filtered glucose load as the eGFR falls, rather than at a sharp pharmacodynamic cut-off; the values of 25 mL/min/1.73 m^2^ for dapagliflozin and 20 mL/min/1.73 m^2^ for empagliflozin reflect the lower bound of trial inclusion and current regulatory indication rather than a pharmacodynamic cut-off below which the agent is inactive, and the hemodynamic and neurohormonal effects are plausibly preserved at lower values [43]. DAPA-RESIST (*n* = 61) compared dapagliflozin with metolazone in loop-diuretic-resistant acute heart failure; the trial is discussed in detail in Section 4.4 [44].

Sotagliflozin is a dual sodium–glucose cotransporter 1 (SGLT1)/SGLT2i with approximately 20-fold in vitro selectivity for SGLT2 (IC50 ~1.8 nM) over SGLT1 (IC50 ~36 nM) [45]. At its therapeutic dosing SGLT2 is fully inhibited in the renal proximal tubule while intestinal brush-border SGLT1, and to a lesser degree cardiac SGLT1, is meaningfully engaged. SGLT1 is expressed in human cardiomyocytes at biologically relevant levels [45]. In experimental models, its cardiac inhibition reduces reactive oxygen species generation and may attenuate pathological glucose uptake driving hypertrophic remodeling, though these remain mechanistic hypotheses [45,46]. Renal SGLT2 inhibition by sotagliflozin produces the same proximal sodium and glucose effects as a selective SGLT2i. Absolute oral bioavailability of sotagliflozin is approximately 25%, with enterohepatic circulation estimated to contribute about half of overall systemic exposure; the terminal half-life is 21–35 h, supporting once-daily dosing. The SOLOIST-WHF trial randomized 1222 patients with type 2 diabetes and recent worsening HF to sotagliflozin or placebo initiated before or within 3 days of discharge, against a planned enrollment of approximately 4000; the trial was terminated early after sponsor withdrawal of funding, and the primary endpoint was revised to a total-event composite per the published statistical analysis plan [47]. Sotagliflozin reduced total (first and recurrent) cardiovascular death, HF hospitalization, and urgent HF visits (51.0 vs. 76.3 events per 100 patient-years; HR 0.67, 95% CI 0.52–0.85; *p* < 0.001) [47]. The drug is currently approved by the US Food and Drug Administration for the reduction in cardiovascular death, HF hospitalization, and urgent HF visits in adults with heart failure across the full LVEF range and irrespective of diabetes status and separately in adults with type 2 diabetes, chronic kidney disease, and additional cardiovascular risk factors. The non-diabetic HF indication extends beyond the T2DM-only SOLOIST-WHF enrollment on the basis of class consistency with the SGLT2i outcomes evidence; sotagliflozin has not received EMA authorization for HF at the time of writing, and availability is therefore regionally restricted. The labeled regimen is 200 mg once daily after correction of volume status, with up-titration after at least two weeks to 400 mg once daily as tolerated; in decompensated heart failure, dosing may begin once the patient is hemodynamically stable, including during hospitalization or at discharge.

Within the SNB framework, SGLT2is function differently from agents such as metolazone or acetazolamide that are added for direct natriuretic effect. Their tubular natriuretic contribution during acute decompensation is modest and inconsistent across trials, consistent with the small and variable increases in urinary sodium excretion observed in mechanistic studies [42,48]. The principal benefit lies in modulation of the hormonal and hemodynamic environment that sustains sodium avidity, with reduced probability of developing diuretic resistance in patients on long-term loop therapy. In a post hoc analysis of EMPEROR-Preserved, empagliflozin was associated with a higher probability of loop diuretic discontinuation, a higher probability of de-escalation, and a lower probability of dose escalation among patients receiving diuretic therapy at baseline [49]. Directionally similar diuretic-sparing patterns have been reported in DELIVER post hoc analyses, although the specific effect sizes are not directly comparable to those of [49]; in EMPEROR-Reduced specific loop diuretic dose changes were not reported, although efficacy on the primary outcome was consistent across baseline diuretic strata [49]. Initiating an SGLT2i before or during hospitalization for acute decompensation is safe and is associated with reduced all-cause mortality and HF rehospitalization at 90 days in meta-analyses of early initiation trials [48].

### 2.7. Vasopressin V2 Receptor Antagonists: Tolvaptan

Tolvaptan competitively blocks the V2 receptor in collecting duct principal cells, preventing ADH-mediated aquaporin-2 insertion into the apical membrane. The consequence is aquaresis, the excretion of electrolyte-free water with minimal accompanying sodium loss. Electrolyte losses relative to the water cleared are negligible; this property distinguishes tolvaptan from every other agent in the SNB armamentarium and explains both tolvaptan’s specific utility and its failure in broader decongestion trials. Its oral bioavailability is approximately 56%, its half-life is 12 h, and its metabolism is predominantly hepatic via CYP3A4, with no meaningful renal elimination adjustment required [5].

The reliance on CYP3A4 imposes several practical constraints. Strong CYP3A4 inhibitors raise tolvaptan plasma concentrations severalfold and are contraindicated, both because of toxic accumulation and because the exaggerated aquaretic response may correct serum sodium more rapidly than safe thresholds permit. Strong inducers reduce tolvaptan systemic exposure substantially, with concomitant attenuation of the aquaretic response [5]. Tolvaptan is a P-glycoprotein inhibitor and produces a modest rise in digoxin exposure; digoxin monitoring and consideration of dose reduction are appropriate when the two drugs are combined, per the prescribing information [5]. Co-administration with an intravenous hypertonic saline solution (HSS) is discouraged: the two agents act through independent mechanisms (free-water excretion versus sodium loading), and their simultaneous use can raise serum sodium at rates well beyond the standard 10 mmol/L per 24 h ceiling (8 mmol/L in patients at elevated osmotic-demyelination risk), with the attendant risk of osmotic demyelination. When tolvaptan is added to a background of loop diuretics, the combined aquaretic–natriuretic effect can be larger than anticipated, particularly in elderly patients or in those with limited free-water intake; intravascular volume, serum sodium, and renal function should be checked at 6–8 h and again at 24 h after the first dose [5,50,51]. The tolvaptan label for hyponatremia carries a boxed warning (FDA) with comparable safety language in the European SmPC (the EMA does not use a formal “boxed warning” category) that the drug should be initiated and re-initiated only in a hospital setting with close serum sodium monitoring and that treatment of hyponatremia should not exceed 30 days because of the risk of hepatic injury observed at higher cumulative exposure. The hepatotoxicity signal originated from long-term experience with the higher-dose tolvaptan formulation used for autosomal dominant polycystic kidney disease but informs the labeled duration in the heart-failure indication. The label also restricts use in hypovolemic hyponatremia, where the dilutional rationale does not apply.

The EVEREST program, two short-term Clinical Status Trials and the parallel Outcome Trial on 4133 patients hospitalized for worsening heart failure in total, randomized tolvaptan or placebo added to standard diuretic therapy. Short-term improvements in dyspnea and body weight were present; all-cause mortality and the cardiovascular death/HF hospitalization composite at 9.9 months were unaffected [50,51]. The neutral result is consistent with the interpretation that sodium-retention-driven congestion dominates in the unselected ADHF phenotype, with vasopressin-mediated free-water retention contributing a secondary component; the trial was not designed to formally separate these contributions.

Tolvaptan’s clinical role is confined to dilutional hyponatremia with persistent congestion. The labeled indication specifies clinically significant hypervolemic or euvolemic hyponatremia with serum sodium < 125 mmol/L or symptomatic milder hyponatremia resistant to fluid restriction; the operational threshold of <130 mmol/L applied in heart-failure practice falls within this criterion in symptomatic patients. In this phenotype, thiazide-type agents are hazardous (worsening hyponatremia via non-osmotic vasopressin potentiation), loop diuretics alone are insufficient, and the dominant mechanism is vasopressin-mediated, collecting duct impermeability rather than primary sodium avidity. The 3T trial [52] included tolvaptan as the V2-antagonist comparator alongside two thiazide-type agents and is discussed in Section 4.2. In patients with serum sodium < 130 mmol/L, tolvaptan 15 mg once daily can be titrated to 30 or 60 mg based on the daily sodium response; correction of hyponatremia should not exceed 10 mmol/L per 24 h in standard-risk patients, with a more conservative ceiling of 8 mmol/L per 24 h in patients at elevated risk for osmotic demyelination: alcoholism and malnutrition, advanced liver disease, and chronic hypokalemia or long-standing hyponatremia [5,50,51]. In heart failure, where the underlying mechanism is dilutional rather than depletional and high-risk features are generally absent, this complication is uncommon. Outside the dilutional-hyponatremia phenotype, the evidence base does not support routine tolvaptan use in SNB protocols.

### 2.8. Adenosine A1 Receptor Antagonists

Adenosine regulates renal microcirculation through A1 receptors on afferent arterioles: macula densa NaCl delivery activates A1 receptors, constricting the arteriole and reducing the GFR. This tubuloglomerular feedback limits distal sodium delivery and protects against hyperfiltration. In heart failure, endogenous adenosine levels are elevated, contributing to the GFR reduction characteristic of the cardiorenal syndrome and compounding loop diuretic resistance by curtailing tubular drug secretion. The mechanistic rationale for A1 antagonism was clear: blocking afferent arteriolar constriction should preserve the GFR, increase tubular drug delivery, and enhance natriuresis [53].

Rolofylline (KW-3902) was the most advanced agent in the class. PROTECT Pilot, the phase II dose-finding study, showed dose-dependent improvements in diuretic response and attenuation of creatinine rise [54]. The subsequent phase III PROTECT trial randomized 2033 patients hospitalized with acute heart failure and renal dysfunction to rolofylline 30 mg intravenously or placebo for three days. The primary trichotomous composite endpoint (treatment success, no change, treatment failure) was unaffected. Worsening renal function occurred in 15.0% versus 13.7%; 60-day mortality and rehospitalization were indistinguishable from placebo [53]. Seizures occurred only in the rolofylline arm, consistent with the predicted central A1 class effect. The program was terminated and no A1 antagonist has subsequently entered late-phase cardiovascular development.

### 2.9. Emerging Agents: Gut-Targeted Sodium Reduction and Beyond

SNB, as conventionally understood, is confined to the nephron. Modulation of sodium balance upstream, at the enterocyte, represents a different therapeutic strategy. The sodium/hydrogen exchanger isoform 3 (NHE3) on the apical membrane of enterocytes accounts for most of the dietary sodium absorption throughout the gastrointestinal tract. Tenapanor is a first-in-class, minimally systemic NHE3 inhibitor designed to act exclusively in the gut; its molecular weight (approximately 1145 Da) and polar surface area are deliberately engineered to restrict systemic absorption to negligible levels [55,56]. Intestinal NHE3 blockade reduces dietary sodium absorption by 20–50 mmol/day in healthy-volunteer and CKD studies across the clinical dose range [55,56], diverting sodium to the stool. Tenapanor currently holds FDA approval for IBS with constipation and for hyperphosphatemia in dialysis patients. No published randomized trial has yet evaluated it in heart failure volume overload, and higher doses carry a diarrhea burden that would complicate use in this population. Clinical evaluation in heart failure, particularly in advanced CKD where renal-targeted diuretics are of limited value, would be needed before any role can be defined.

Beyond tenapanor, investigational approaches target resistance at a pathway level rather than at individual transporters. WNK kinase inhibitors acting on the WNK1/WNK4-SPAK/OSR1-NCC cascade, the molecular substrate of NCC upregulation during the braking phenomenon, could theoretically achieve more selective distal blockade than thiazides; preclinical rodent data support this rationale [57], and we are aware of no clinical-stage WNK inhibitor that has reached late-phase cardiovascular evaluation at the time of writing. Direct ENaC blockade for proteolytic ENaC activation in nephrotic syndrome (Section 2.4) is mechanistically rational but lacks confirmatory trial data in heart-failure-associated nephrotic edema. The pharmacological properties of the diuretic agents discussed in this section are summarized in Table 1.

## 3. The Biology of Diuretic Resistance: From Pharmacokinetics to Molecular Adaptation

Diuretic resistance is a clinical label applied to several overlapping phenomena that share a common presentation, have inadequate natriuresis despite high loop diuretic doses, but differ in their dominant mechanism. When the label is treated as a single entity, dose escalation tends to address few of these mechanisms specifically. No universally accepted quantitative definition exists. Operational thresholds proposed in the literature combine inadequate natriuretic response to cumulative high-dose intravenous furosemide over 24–72 h, with specific cut-offs varying across protocols [3,21].

### 3.1. Pharmacokinetic Mechanisms

Loop diuretics must achieve a minimum luminal concentration at the apical surface of TALH cells to produce measurable natriuresis. That threshold rises substantially with the degree of renal sodium avidity, and in decompensated heart failure the shift can be marked [21]. The delivery pathway is active secretion by basolateral OAT1 and OAT3 in proximal tubular cells; passive filtration contributes minimally because more than 95% of the drug is protein-bound. This secretory step is competitively saturated by accumulated uremic organic anions at concentrations already elevated in CKD stage 3 and rising with declining GFR [5]. In advanced CKD with concurrent decompensation, this competitive inhibition is sufficient to shift an otherwise adequate dose below the tubular threshold for natriuresis. Dose escalation raises plasma drug concentrations and partially overcomes the secretory bottleneck but does so inefficiently relative to addressing the upstream mechanism.

The wide and unpredictable oral bioavailability of furosemide (Section 2.1) is one reason intravenous administration is the recommended route during in-hospital decongestion (ESC 2021, class I [35]). Hypoalbuminemia below approximately 20 g/L adds a further layer: non-esterified fatty acids accumulate in this state and compete with furosemide at the basolateral OAT1/OAT3 transporters, reducing tubular drug secretion despite an increased unbound plasma fraction. For example, in nephrotic syndrome an additional component is intratubular sequestration of furosemide by filtered albumin [5].

The distinction between pharmacokinetic resistance and pharmacodynamic resistance has important clinical implications. In one, insufficient drug reaches the tubular lumen; in the other, the drug reaches the lumen but the tubule fails to respond. In patients with chronic loop diuretic exposure, blunted natriuresis predominantly reflects reduced tubular response; the earlier emphasis on pharmacokinetic barriers has been progressively refined as the molecular basis of the braking phenomenon has been clarified [5,21]. Urinary furosemide excretion measured alongside natriuresis separates the two components, but the assay is a research tool rather than a routine clinical option; in practice the distinction is inferred from response patterns and dose-titration behavior.

### 3.2. The Braking Phenomenon: Molecular Biology

The braking phenomenon is the structural adaptation of the DCT to chronic sodium loading from the loop. A single loop diuretic dose increases NaCl concentration at the early DCT by approximately threefold to fourfold; with repeated dosing over days to weeks, DCT cells undergo hypertrophy and proliferation, with parallel upregulation of NCC at the apical membrane. Rodent morphometric studies document two- to threefold increases in DCT fractional cortical volume, amplification of the basolateral cell membrane, and a measurable extension of total DCT length; in humans, indirect evidence from urinary exosome NCC abundance and limited biopsy series supports the same direction of effect [5].

The molecular mechanism of NCC upregulation during the braking phenomenon runs through the WNK-SPAK/OSR1-NCC phosphorylation cascade. WNK1 and WNK4, the dominant DCT isoforms, act as intracellular chloride sensors: when intracellular Cl^−^ falls, as occurs when NaCl delivery from the loop is high and NCC cannot keep pace, WNK kinases are activated, phosphorylate SPAK and OSR1, and through them phosphorylate NCC, increasing its activity and promoting its trafficking to the apical membrane, raising functional NCC density along the DCT [57,58,59,60,61].

Intracellular chloride concentration is the regulatory node sensed by WNK kinases, and it links several apparently unrelated clinical observations. During chronic loop diuretic therapy, high distal sodium delivery depletes intracellular Cl^−^ in DCT cells, keeping WNK kinases active and NCC upregulated. Hypochloremia from combined loop-and-thiazide therapy reaches the same cascade through a different entry point: low circulating Cl^−^ reduces tubular delivery and depresses intracellular Cl^−^. In potassium deficiency, Kir4.1-mediated Cl^−^ efflux from DCT cells lowers intracellular chloride independently [58,59]. Chronic loop diuretic exposure, hypochloremia, and potassium deficiency converge on the same final pathway; the physiological corollary is that correction of K and Cl deficits should accompany distal blockade in decompensated heart failure for optimal natriuretic yield, an inference that is mechanistically reasoned rather than trial-validated [62].

### 3.3. Post-Diuretic Sodium Retention

After each dose of a short-acting loop diuretic, renal sodium reabsorption rises above the baseline across the distal nephron, with a smaller proximal contribution. Acute volume contraction during the natriuretic phase briefly activates RAAS and sympathetic pathways; the shorter the diuretic half-life, the longer the relative duration of this counter-regulatory phase within the dosing interval. Furosemide’s natriuretic action lasts approximately 3–6 h; once-daily dosing leaves 16–21 h of post-diuretic sodium retention during which dietary intake accumulates unchecked. Daily natriuretic balance, drug-induced loss minus post-diuretic retention minus dietary intake, can be substantially negative even at high furosemide doses when dosing frequency is inadequate [3,21].

### 3.4. The Third Compartment: Tissue Sodium and Glycosaminoglycan Biology

The standard two-compartment model of sodium distribution, intravascular and interstitial with renal excretion as the sole regulatory output, is an incomplete description of sodium homeostasis. Work developed principally by Titze and colleagues over two decades indicates that skin, muscle, and myocardium can sequester sodium with a degree of osmotic inactivation through binding to negatively charged glycosaminoglycan (GAG) networks in the extracellular matrix, although the dynamics and physical basis of this third compartment remain debated [63,64,65]. In humans, ^23^Na magnetic resonance imaging has quantified tissue sodium in skin and skeletal muscle; concentrations are elevated in heart failure, CKD, and hypertension and correlate with markers of cardiovascular remodeling and adverse prognosis [63,66,67].

The neurohormonal environment of HF may disrupt GAG sulfation and cross-linking, reducing tissue binding capacity and shifting osmotically inactive sodium toward the active interstitial pool. This could contribute to the disproportionate edema seen when sodium balance is only modestly positive and to incomplete decongestion despite substantial urinary sodium losses [63,64,66]. At present, tissue sodium imaging remains a research tool.

### 3.5. Neurohormonal Amplification

Reduced effective arterial blood volume in heart failure activates RAAS and sympathetic pathways in a self-reinforcing loop. Angiotensin II directly stimulates NHE3 in the proximal tubule, increasing proximal reabsorption and reducing sodium delivery to the TALH, which shrinks the substrate available for NKCC2 inhibition even when luminal drug concentrations are adequate. Aldosterone upregulates ENaC and Na-K-ATPase in the collecting duct, adding a distal component of sodium retention independent of DCT hypertrophy and one that mineralocorticoid antagonists specifically address [5]. Angiotensin II also activates WNK4 independently of intracellular chloride, providing a second route to NCC upregulation that operates even when distal sodium delivery has not been chronically elevated [57].

### 3.6. Diuretic Efficiency: A Composite Metric

Diuretic efficiency, weight loss per unit of loop diuretic administered (kg per 40 mg furosemide equivalent), was introduced by Testani and colleagues as a composite metric that integrates the major resistance mechanisms operating at the nephron [68]. Low efficiency reflects pharmacokinetic barriers to tubular delivery, distal tubular adaptation via the WNK–SPAK–NCC axis, and the neurohormonal sodium avidity that compounds both; third-compartment buffering contributes an additional component that the metric cannot resolve directly. In a prospective analysis [68], diuretic efficiency correlated with all-cause mortality and rehospitalization at 6 months more strongly than absolute diuretic dose. The measurement requires no additional laboratory work. The formula gives a bedside-computable indicator that urine output or weight change alone does not provide. Paired with 2 h spot urinary sodium, the two metrics are complementary: urinary sodium reflects tubular transport capacity, diuretic efficiency reflects net fluid removal relative to drug burden, and they can dissociate in patients with high dietary intake or active third-compartment sodium mobilization.

## 4. Sequential Nephron Blockade: Clinical Evidence

### 4.1. Rationale

If the dominant obstacle to further natriuresis is compensatory sodium reabsorption at a site downstream from the loop, an agent that inhibits that transporter should restore net excretion. The most studied combination is loop plus thiazide or thiazide-like agent, targeting the braking-phenomenon-driven hypertrophy of the DCT. Acetazolamide acts upstream, increasing proximal sodium delivery to both the loop and the distal tubule; the two strategies are not mutually exclusive [6,39].

### 4.2. Loop Plus Thiazide-Type Diuretics

Loop-plus-thiazide combinations entered clinical use at least three decades before adequately powered randomized trials became available. Cox and colleagues (the 3T trial) compared metolazone 5 mg twice daily, intravenous chlorothiazide 500 mg twice daily and oral tolvaptan 30 mg once daily as adjuncts in 60 patients with loop diuretic resistance defined as inadequate response to high-dose intravenous furosemide (entry threshold ≥ 240 mg/day; mean enrolled dose 612 ± 439 mg/day) [52]. Forty-eight-hour weight loss and cumulative urine output did not differ across arms; the trial was underpowered for non-inferiority and unselected for hyponatremia and serves primarily to anchor the three augmentation strategies on a common population rather than to discriminate between them [52]. A retrospective cohort study comparing metolazone and intravenous chlorothiazide across 220 hospital encounters found a borderline difference in median 24 h net urine output of uncertain clinical significance, with chlorothiazide substantially more expensive; these data contribute to the predominance of oral metolazone in clinical practice [15].

CLOROTIC was the first adequately powered randomized trial of thiazide augmentation. In 230 patients hospitalized with acute heart failure, hydrochlorothiazide (dosed by renal function at randomization: eGFR > 50, 25 mg/day; eGFR 20–50, 50 mg/day; eGFR < 20, 100 mg/day, with adjustment permitted only if the eGFR changed during treatment) added to intravenous furosemide increased weight loss at 72 h and improved the rate of successful decongestion at both 72 and 96 h [69]. A post hoc analysis found no significant interaction across the ejection fraction spectrum (HFrEF, HFmrEF, HFpEF), although the HFmrEF stratum was small (*n* = 17) [70]. A separate post hoc analysis stratified by the eGFR showed a graded attenuation in the point estimate of the weight-loss benefit as renal function declined, reported as the adjusted weight difference at 72 h [69]. The original authors described the response in the eGFR ≥ 60 stratum as significantly greater than in the lower strata, although on standard inferential interpretation the upper bound of every stratum CI crosses zero and the interaction term is not significant; the practical reading is of a quantitatively attenuated, but not formally eliminated, weight-loss signal as the eGFR falls. An increase in creatinine of at least 26.5 µmol/L or a fall in the eGFR of more than 50% occurred in 46.5% of HCTZ-treated patients versus 17.2% of placebo, an imbalance that qualifies the net benefit in patients with baseline renal impairment [69]. A meta-analysis of available randomized data confirmed improvements in weight loss, urine output, and diuretic efficiency; worsening renal function events did not differ between groups, while hypokalemia rates were higher in combination arms [71].

### 4.3. Acetazolamide

A prespecified natriuresis analysis of ADVOR by Verbrugge and colleagues showed that allocation to acetazolamide on a standardized loop diuretic background independently increased urinary sodium concentration and total natriuresis, consistent with proximal sodium delivery facilitating the sodium load available for inhibition at loop-treated segments [6,40]. ADVOR was the first adequately powered randomized placebo-controlled trial of this combination. In 519 patients on standardized background intravenous loop diuretics, acetazolamide 500 mg intravenously daily for up to three days or until complete decongestion raised the proportion achieving successful decongestion at day three from 30.5% to 42.2% without significantly increasing worsening renal function or electrolyte disturbance rates [39]. The secondary endpoint of death from any cause or rehospitalization for heart failure at three months occurred in 29.7% of the acetazolamide group versus 27.8% of placebo, indicating that the decongestion advantage did not translate into a detectable effect on hard clinical outcomes within the trial’s follow-up window [39]. A second potentially relevant mechanism beyond proximal sodium delivery is the correction of the hypochloremic metabolic alkalosis that accompanies sustained loop and thiazide therapy, discussed in Section 2 and proposed as an independent contributor to diuretic resistance [6,72].

### 4.4. SGLT2 Inhibitors as Adjunctive Decongestion Agents

While acetazolamide acts on the proximal tubule, SGLT2is influence sodium handling across the proximal–distal axis through a combination of tubular and neurohormonal mechanisms. EMPULSE randomized 530 patients hospitalized for acute heart failure to empagliflozin or placebo initiated in-hospital after clinical stabilization (median time from admission to randomization 3 days), irrespective of ejection fraction or diabetic status. The hierarchical composite of death, recurrent heart failure events, and health status change favored empagliflozin at 90 days [43]. A subsequent analysis of EMPULSE addressing decongestion outcomes showed improved diuretic efficiency and fewer residual congestion markers in the empagliflozin arm [73].

DAPA-RESIST asked whether dapagliflozin could serve as an alternative to metolazone in patients with documented loop diuretic resistance. In 61 patients, dapagliflozin 10 mg daily produced weight change at 96 h not significantly different from metolazone 5–10 mg daily, with fewer episodes of hypokalemia and hyponatremia [44]. The cumulative dose of background intravenous furosemide required to reach this comparable weight change was substantially higher in the dapagliflozin arm, and loop diuretic efficiency was numerically lower with dapagliflozin. Fluid removal was therefore comparable per patient but inferior per loop diuretic dose: dapagliflozin matched metolazone only at the cost of higher loop exposure and is not a per-dose equivalent substitute. The trial was not designed for non-inferiority, and the sample of 61 patients is too small to detect clinically meaningful differences in hard outcomes, so the results are best interpreted as hypothesis-generating, with a more favorable electrolyte profile for dapagliflozin. The mechanistic basis for fewer electrolyte disturbances is that SGLT2 inhibition does not deliver additional sodium to collecting duct ENaC and does not drive the kaliuresis that thiazides produce [42].

The acute diuretic efficiency advantage of SGLT2i over placebo, when pooled across trials, does not reach statistical significance; individual trial results are heterogeneous [43,48,54]. In patients with established loop diuretic resistance, these agents are not equivalent in decongestive potency to thiazide-type agents; their role in acute decompensation is better understood as a modulation of the neurohormonal context governing tubular sodium avidity. A recent meta-analysis of eleven randomized trials encompassing 7517 patients is consistent with this dissociation. In this cohort the combination diuretic strategies improved daily urinary output, weight loss, and diuretic efficiency without an effect on all-cause mortality or heart failure events, whereas in-hospital SGLT2i initiation reduced heart failure events and worsening renal function [74].

Whether acetazolamide retains an incremental decongestive effect in patients already on an SGLT2i, now widely used as guideline-directed therapy in eligible HFrEF, remains open. ADVOR excluded such patients and DEA-HF was underpowered to adjudicate the question (Section 2.5), and no adequately powered randomized trial has tested the combination in acute decompensation. Pending such evidence, acetazolamide addition in SGLT2i-treated patients is reasonable when the background loop dose remains at or below the ADVOR range and hypochloremic alkalosis is present, provided advanced uremic acidosis has been excluded. The key randomized trials informing sequential nephron blockade strategies in acute heart failure are summarized in Table 2.

## 5. Natriuresis-Guided Therapy

Managing volume overload without a real-time metric of diuretic response relies largely on indirect indicators. Body weight changes within the first 24 h are informative but imprecise; clinical signs of decongestion lag behind the hemodynamic response. Spot urinary sodium measured 2, 6, or 12 h after the first dose correlates with subsequent natriuresis and identifies inadequate responders before clinical signs become manifest [3]. *The European Journal of Heart Failure* expert consensus on natriuresis-guided diuretic titration consolidates the operational thresholds applied throughout this section, recommending early intensification when spot urinary sodium falls below 50 mmol/L (expert consensus threshold) or 70 mmol/L (PUSH-AHF trial trigger) at two hours or urine output below 100–150 mL/h at six hours after the first intravenous loop dose [14,77].

Sodium measurement on a spot urine sample is preferable to 24 h collection in the acute setting for several reasons. Results are available within the first two hours after the diuretic dose, a window that matches the pharmacodynamic peak of intravenous furosemide and permits step-by-step dose adjustment rather than a retrospective audit of the previous day’s therapy [3,5]. The practical burden on the ward is considerably lower, and the value obtained reflects the tubular response to the drug administered. A 24 h collection averages across dosing intervals, periods of inadequate response, and changes in intake and is rarely feasible in patients with frequent voiding or incontinence; its role is confined to subsequent verification of cumulative natriuresis, while bedside decision-making relies on spot measurements [3].

A related diagnostic instrument is the furosemide stress test (FST), introduced by Chawla and colleagues and validated in prospective multicenter cohorts [80,81]. A single intravenous bolus of furosemide (1.0 mg/kg in loop-naive patients, 1.5 mg/kg in those with prior exposure) is administered, and urine output is measured hourly for six hours. A 2 h output below 200 mL identifies patients with limited tubular functional reserve and predicts progression to stage 3 acute kidney injury (AKI) with an area under the curve of approximately 0.87 [80]. The test was developed for early AKI risk stratification; extrapolation to the heart failure population requires caution, the hypovolemic response to a high-dose bolus can be clinically significant, and volume replacement may be needed during the test period. By analogy rather than formal validation in HF, a functional challenge of this kind can help distinguish an intact but downregulated nephron, likely to respond to combination therapy, from a severely injured tubular epithelium in which further escalation yields diminishing returns and additional risk. In the validation cohort by Koyner and colleagues, 2 h urine output after FST showed comparable or superior discrimination to urinary biomarkers of tubular injury for progression to stage 3 AKI and for need for renal replacement therapy, though head-to-head prospective comparison in larger cohorts is not yet available [81].

PUSH-AHF tested whether a formal algorithm using a spot urinary sodium threshold of <70 mmol/L to trigger escalation or combination therapy would outperform standard care. The threshold was a pragmatic trial trigger rather than a universal definition of inadequate response. In 310 patients, the natriuresis-guided arm achieved higher 24 h urinary sodium, while all-cause mortality or HF rehospitalization at 180 days was 31% in both arms [77]. The neutral 180-day result is consistent with the trial design: the natriuresis-guided algorithm was applied only during the first 36 h of hospitalization. A sustained effect at 180 days would require either that early decongestion alone prevents late events (plausible but unproven) or that the algorithm be extended throughout the decongestion phase rather than confined to the initial window. A prespecified interaction analysis showed treatment effect heterogeneity across eGFR strata, with a numerically more favorable hazard ratio at a lower eGFR; this hypothesis-generating observation from a subgroup analysis within a globally neutral primary outcome warrants prospective confirmation before influencing practice [78]. The 2021 ESC heart failure guidelines indicate that early urinary sodium assessment may be considered to evaluate diuretic response [35]; PUSH-AHF supports this approach and shows that algorithm-driven escalation improves the short-term biochemical endpoint without an increase in worsening renal function or adverse-event rates within the trial’s power, while the neutral 180-day outcome limits the strength of any broader inference [78].

A post hoc analysis of PUSH-AHF stratified patients by baseline BAN-ADHF (diuretic-resistance risk) score [76]. Patients at high diuretic-resistance risk showed lower 24 and 48 h natriuresis and a higher risk of the composite endpoint at baseline; natriuresis-guided therapy increased 24 h natriuresis consistently across diuretic-resistance strata, suggesting that the algorithm may add the most value where standard care is most likely to fail [76].

## 6. A Practical Approach to Refractory Congestion

The framework that follows synthesizes published trial evidence, ESC and Kidney Disease: Improving Global Outcomes (KDIGO) guidance, label-approved dosing, and physiological reasoning where direct prospective data are lacking. It has not been tested against a fixed-sequence combination protocol in an adequately powered randomized trial, and its components carry heterogeneous levels of evidence. Monitoring thresholds and urinary sodium triggers (Table 3a) are largely guideline- and trial-backed; phenotype-based adjunct selection (Table 3b) is a hypothesis-generating synthesis of mechanistic and post hoc trial data; agent-by-eGFR choices (Table 3c) blend prospective evidence with pharmacokinetic extrapolation where CKD-stratified trials are unavailable; electrolyte targets (Table 3d) reflect physiological rationale except where explicitly noted. The evidence source of each row is annotated. The framework is offered as a structured aid to clinical reasoning.

### 6.1. Before the First Dose: Establishing Baseline and Dosing Correctly

A baseline transthoracic echocardiogram, recommended at AHF admission by current guidance [35], also informs SNB by characterizing right ventricular function and the preload sensitivity that affects tolerance of layered diuresis. Echocardiography supports the assessment of congestion at the bedside. Finding of an inferior vena cava diameter greater than 21 mm identifies patients at higher risk of adverse outcome independent of left ventricular ejection fraction [86]. Distinguishing heart failure with reduced preserved ejection fraction informs subsequent agent choice, since preload-sensitive HFpEF with restrictive filling tolerates aggressive thiazide addition less well than HFrEF, and severe right ventricular dysfunction or tricuspid regurgitation alter both the threshold for ultrafiltration and the interpretation of venous congestion scores [87].

Before any diuretic, the following are collected: serum Na, K, Cl, HCO_3_, Mg, and creatinine, urea, and a spot urinary sodium [3,14,21]. Pre-dose urinary sodium provides the reference value against which the 2 h post-dose measurement acquires meaning, and its absence limits interpretation. The patient is weighed on a calibrated scale after voiding, in comparable clothing, at a consistent time; daily weight remains the most informative non-laboratory measure of net fluid balance [3].

The intravenous starting dose is determined from the maintenance oral regimen. The oral-to-IV furosemide conversion is approximately 2:1, reflecting mean oral bioavailability near 50% under euvolemic conditions. In decompensation with probable gut edema, actual bioavailability may be 20–30% or less. The high-dose arm of the DOSE trial (2.5× the outpatient oral dose given intravenously) produced greater short-term decongestion and symptom improvement than the 1× strategy at the cost of a transient creatinine rise [75]; starting at this multiplier is pharmacokinetically appropriate in patients with a low eGFR or an elevated urine albumin-to-creatinine ratio, where OAT-mediated tubular secretion is further compromised. For class-to-class conversions, torsemide 20 mg ≈ furosemide 40 mg ≈ bumetanide 1 mg [5]. Twice-daily bolus dosing is generally preferred for furosemide and bumetanide given their short half-life, for the reasons developed in Section 3.3; continuous infusion is an alternative supported by the DOSE trial as broadly equivalent to bolus dosing [75].

### 6.2. At 2 Hours: The First Decision Point

Spot urine is collected at exactly 2 h after the intravenous bolus, and urinary sodium is measured. The 2 h value correlates with the 6 h natriuretic response in prospective cohort data [68], providing a rational basis for early escalation; PUSH-AHF subsequently used this threshold as the operational trigger [77]. The threshold adopted as an operational reference in this review is the PUSH-AHF protocol trigger of <70 mmol/L, as it is the only threshold tested in a randomized trial [77]. A more aggressive dose-doubling threshold of <50 mmol/L has been proposed in expert consensus [3,14] and may be applied where centers prefer earlier escalation; the two values are not interchangeable and reflect different protocols and endpoints. Values > 70 mmol/L are consistent with a natriuretic response unlikely to benefit from further immediate escalation.

Three conditions alter interpretation. In acute tubular necrosis, urinary sodium is paradoxically high despite oliguria; high urinary sodium from tubular dysfunction should not be confused with an adequate natriuretic response. In patients on SGLT2i, glucose-driven osmotic diuresis dilutes urinary sodium concentration independently of tubular sodium avidity; urinary sodium is interpreted in the context of urine glucose and urine output. A third modifier acts on drug delivery rather than tubular response: furosemide secretion is progressively impaired below a serum albumin of approximately 20 g/L (Section 3.1). Albumin–furosemide co-infusion has been proposed as a rescue option in selected patients unresponsive to standard intensification, although randomized evidence remains limited and most guidelines do not support its routine use [3].

### 6.3. When Escalation Fails: The Combination Threshold

When successive doublings of the loop diuretic dose fail to raise the 2 h urinary sodium above the natriuresis threshold (Section 6.2), further loop escalation is unlikely to help, and the reasoning shifts to which mechanism of resistance predominates. The biochemical pattern at this point can be informative, although the mappings that follow are physiological inferences rather than prospectively validated rules: hypochloremic metabolic alkalosis with hypokalemia and high urinary sodium in the face of adequate urine output is consistent with the braking phenomenon (distal NCC upregulation) and argues for a thiazide-type agent; metabolic alkalosis without prominent sodium escape, particularly in a patient with a relatively low chronic loop diuretic burden (oral dose ≤ 80 mg furosemide equivalent/day, close to the ADVOR enrolled range), argues for acetazolamide; low urinary sodium despite adequate loop dosing in a patient with dilutional hyponatremia and serum chloride < 96 mmol/L is consistent with chloride depletion as the primary driver of sodium avidity [6,72]. These phenotypes frequently coexist in advanced decompensation.

Because these patterns overlap far more often than they occur in isolation, the clinician needs an order of priority rather than a list. The following sequence follows from the physiology and from the safety data reviewed in Section 7; it has not been tested prospectively and is offered as reasoned opinion. Potassium and magnesium are corrected before or alongside any adjunct, because distal blockade increases kaliuresis and because potassium replacement remains ineffective while serum magnesium is low. Chloride repletion belongs in the same first step when serum chloride is below 96 mmol/L, since chloride depletion sustains both WNK-SPAK activation and renin release and will otherwise blunt the response to whichever agent is chosen. When proximal and distal features coexist, the abnormality that is both more severe and easier to follow is treated first. Hypochloremic alkalosis with a pre-dose urinary sodium below 20 mmol/L and a relatively low chronic loop burden, the pattern set out in Table 3b, favors acetazolamide, whose effect can be read directly from the fall in serum bicarbonate; a rebound pattern with preserved urinary sodium after the first hours favors a thiazide-type agent instead. Agents are then sequenced rather than stacked. A second adjunct is added only once the natriuretic response to the first has been measured at 2 h and found insufficient, and a third agent is rarely justified outside specialist supervision, since hypokalemia, hyponatremia and hypotension compound. SGLT2i therapy sits outside this sequence: it is continued or started as foundational treatment and does not count as an escalation step. The geriatric vulnerability phenotype of Table 4 modifies each of these decisions by lowering the starting dose and shortening the interval to reassessment, not by changing the agent.

### 6.4. Thiazide Augmentation: Agent, Dose, and Timing

Metolazone (2.5–5 mg orally, up to 10 mg in severe diuretic resistance), which retains diuretic activity at an eGFR < 30 mL/min/1.73 m^2^ in retrospective data (not yet confirmed in a prospective CKD-stratified trial), is the most widely used thiazide-type agent for SNB in North American practice; hydrochlorothiazide or bendroflumethiazide are more common in European centers (Section 2.2). Chlorthalidone (12.5–25 mg once daily) is a valid alternative for outpatient combination regimens: its half-life of 40–60 h provides sustained distal NCC blockade throughout the day, and pharmacological and observational evidence supports residual activity at an eGFR as low as 15–30 mL/min/1.73 m^2^ (with the strongest prospective data, from the CLICK trial, on the blood pressure endpoint rather than natriuresis specifically); prospective natriuresis-focused CKD-stratified data are lacking [5,11]. Intravenous chlorothiazide (500–1000 mg) produces equivalent diuretic outcomes to metolazone in retrospective comparative data [15] and is appropriate when oral absorption is compromised. Administering metolazone 30–60 min before the intravenous loop bolus is pharmacokinetically rational and operationally straightforward. Available retrospective comparative data do not identify a single optimal interval and do not show a clear efficacy advantage of one timing strategy over another [17,88]; pre-dosing remains a reasonable default on pharmacokinetic grounds (matching the metolazone Cmax with the rise in luminal furosemide), while concurrent administration has been reported with a broadly similar diuretic effect in some series. Intravenous chlorothiazide should not require pre-dosing.

Hyponatremia requires explicit consideration before a thiazide is added. Thiazide-type agents impair free-water excretion primarily through reduced distal tubular dilution, with non-osmotic vasopressin release contributing in a subset of patients, and can precipitate severe symptomatic hyponatremia, with onset typically within 1–4 weeks of initiation and occasional rapid presentations within 24–72 h in older patients, particularly women, and in those with low baseline solute intake [12]. In the context of SNB for ADHF, the background loop diuretic modifies but does not abolish this risk. In patients with a serum sodium below 135 mmol/L at the time of the decision to combine, thiazide addition may be best avoided in favor of an alternative adjunct (acetazolamide, SGLT2i, or tolvaptan when the dominant mechanism is water retention) [12,14]; a serum sodium between 135 and 138 mmol/L warrants closer monitoring (electrolytes at 12 and 24 h after the first combined dose) and a lower starting thiazide dose. Correction of hypokalemia and hypomagnesemia before or in parallel with thiazide initiation reduces, but does not eliminate, the risk [12]. A parallel consideration applies to HFpEF with small, stiff ventricles and restrictive filling, in whom preload reduction from combined loop–thiazide diuresis can produce an abrupt fall in stroke volume; when the echocardiographic profile suggests pronounced preload dependence, initial thiazide doses should be at the lower end of the range and the response reassessed clinically and, where indicated, by imaging before further escalation [87].

### 6.5. Acetazolamide: The Proximal Strategy

Acetazolamide 500 mg intravenously once daily for three consecutive days, on a background of standardized intravenous loop diuretics, is the ADVOR regimen, which applied a fixed acetazolamide dose to all randomized patients regardless of baseline kidney function, with an estimated glomerular filtration rate (eGFR) < 20 mL/min/1.73 m^2^ serving as an exclusion criterion rather than a trigger for dose adjustment [39]. The proposed reduction to 250 mg IV daily in patients with an eGFR < 40 mL/min/1.73 m^2^ represents a pragmatic extrapolation intended to limit the additional acid load in this range of kidney function and was not part of the ADVOR study protocol; rationale and trial-level results are developed in Section 4.3. At the bedside, the expected fall in serum bicarbonate is the intended pharmacodynamic effect and does not warrant stopping the drug unless there is concurrent respiratory failure. The benefit appears most reliable at lower background loop diuretic doses (median home oral furosemide-equivalent dose in ADVOR 60 mg/day, translating per protocol to 120 mg/day intravenously during the trial); in patients already on very high loop doses, the incremental proximal contribution may be less apparent, and patient selection should weigh baseline loop diuretic exposure and concomitant SGLT2i therapy [41].

### 6.6. The Hypochloremia Phenotype

Diuretic resistance in the context of serum chloride < 96 mmol/L has been hypothesized to represent a distinct phenotype, although the supporting evidence is limited. A recent review reframes chloride from a passive electrolyte to a regulator of NCC, NKCC2 and macula densa renin signaling, supporting the rationale for chloride-targeted therapy in the hypochloremic subset [62]. An interaction analysis from the SALT-HF trial (conducted in ambulatory patients with worsening heart failure rather than in hospitalized ADHF) showed an interaction by serum chloride for 3 h natriuresis (p-interaction = 0.008 in a subgroup of 16 hypochloremic patients), with directionally consistent but underpowered signals for the 7-day congestion score and 30-day clinical events; no benefit was seen in normochloremic patients [82]. This is a single post hoc subgroup in a small ambulatory trial, and on its own it cannot support a general recommendation. Co-infusion of a small-volume HSS (100 mL of 3% NaCl over 30–60 min alongside the furosemide bolus) in a patient with serum chloride < 96 mmol/L, hypochloremic alkalosis, and unsatisfactory loop diuretic response is therefore best regarded as an investigational, center-specific strategy pending prospective confirmation rather than as a standard maneuver. Where the dominant abnormality is dilutional hyponatremia rather than isolated hypochloremia, thiazide addition should be avoided because of the risk of worsening hyponatremia via non-osmotic vasopressin potentiation; tolvaptan is the indicated alternative, with small-volume hypertonic saline reserved for severe symptomatic hyponatremia. Where serum sodium is preserved, intravenous potassium chloride corrects hypokalemia and hypochloremia simultaneously and is less controversial than HSS as a chloride-repletion vehicle [72,82].

### 6.7. Monitoring Protocol

The monitoring intensity should match the aggressiveness of the intervention (see Table 3a for the full schedule).

1. Serum Na, K, Cl, HCO_3_, creatinine: every 12 h for the first 48 h of combination therapy then every 24 h until electrolyte targets are stable and decongestion rate is established.
2. Serum magnesium: every 24 h during active combination therapy. Hypomagnesemia (<0.8 mmol/L) renders hypokalemia refractory to supplementation; correct magnesium before or simultaneously with potassium; target ≥ 0.8 mmol/L throughout active diuresis [3,5].
3. Spot urinary sodium: a pre-treatment (T0) baseline value, then at 2 h after each new or escalated dose during the first 24 h, and then once daily (first morning void, before the AM dose) to confirm a sustained natriuretic response.
4. Urine output: documented hourly for the first 12 h of combination therapy, then 6-hourly. Oliguria (<0.5 mL/kg/h for 2 consecutive hours) should prompt urinary sodium and creatinine measurement before diuretic dose reduction to distinguish volume depletion (low UNa, high urine creatinine) from tubular injury (high UNa, low urine creatinine)
5. Body weight: once daily, morning, same scale, and same conditions. The targets are as follows (consistent with Table 3a): 0.5–1.5 kg/24 h as a commonly applied clinical target in guideline-aligned practice; 1–2 kg/24 h in moderate congestion; and up to 2–3 kg/24 h in severe congestion with adequate blood pressure, not exceeding the estimated rate of interstitial-to-intravascular refilling.

### 6.8. Ultrasound Assessment of Congestion: The VExUS Protocol

Deciding when to de-escalate a sequential combination is, in practice, harder than deciding when to begin it. Clinical signs of residual congestion are insensitive, and the daily weight that anchors the early decongestion phase loses information value as decongestion extends beyond 5–7 days, when nutrition and body composition begin to confound it. Natriuretic peptides respond with a delay that does not match the time course of the decongestion phase. The Venous Excess Ultrasound (VExUS) protocol, introduced by Beaubien-Souligny and colleagues, addresses this gap with a semiquantitative integration of four point-of-care ultrasound markers: inferior vena cava (IVC) diameter and Doppler waveforms of the hepatic, portal and intrarenal veins. The protocol grades systemic venous congestion from 0 to 3: grade 0 corresponds to no congestion (IVC < 2 cm or absence of abnormalities); IVC dilation ≥ 2 cm is the prerequisite for grades 1–3, with grade 1 indicating mild and non-critical abnormalities, grade 2 indicating moderate congestion with one severely abnormal waveform, and grade 3 indicating severe congestion with two or more severely abnormal waveforms [89].

The protocol has two roles in the SNB workflow: baseline characterization of the severity of congestion and serial monitoring of its resolution. At admission, a VExUS grade 3 in patients hospitalized for ADHF has been independently associated with higher mortality and with earlier HF rehospitalization [90]. A post hoc observational analysis has also linked higher VExUS grades to lower loop diuretic efficiency, irrespective of baseline creatinine, suggesting that systemic venous pressure itself contributes to the resistance phenotype [91]. These observations position VExUS primarily as a prognostic marker that may inform the intensification or de-escalation of combination therapy; its role as a diagnostic adjunct to physical examination is secondary.

Serial assessments during active decongestion can document the trajectory of venous unloading that clinical examination tends to miss. Normalization of portal vein pulsatility and the return of a phasic intrarenal venous flow pattern often precede the resolution of peripheral edema, and a one-point reduction in VExUS grade before discharge has been explored in observational cohorts as a potential target [90,92], although it is not a validated trigger for SNB escalation or de-escalation. In a single-center randomized controlled trial in patients with type 1 cardiorenal syndrome, VExUS-guided decongestion did not improve renal recovery compared with standard care but significantly increased the probability of achieving adequate decongestion [92]; the trial was not powered for hard outcomes, and the findings should be interpreted as hypothesis-generating.

How the grade should alter drug selection is the question that matters for a personalized strategy, and no trial has tested it. What follows is a proposal consistent with the physiology and with the observational data cited above. A grade 2 or 3 score together with an adequate 2 h natriuretic response argues for continuing the current regimen rather than adding an agent, because the obstacle is the volume still to be removed and not the tubular response. The same score with a natriuretic response below the threshold identifies the patient in whom combination therapy is most likely to repay its electrolyte cost and shifts the balance away from further loop dose escalation. A grade 0 or 1 score in a patient who remains symptomatic with low urinary sodium and rising creatinine raises the opposite concern, that intravascular volume has already been depleted while symptoms persist for another reason, and favors de-escalation of the adjunct rather than intensification. Normalization of portal pulsatility and of intrarenal venous flow while peripheral edema persists suggests that the sodium still to be removed is interstitial rather than intravascular, a situation in which slower continued removal is more appropriate than a further agent. Used in this way, the score acts as a brake or an accelerator on an escalation decision that remains driven by natriuresis, not as an independent trigger.

When the standard four-point examination is precluded by obesity, bowel gas, or surgical dressings, the extended eVExUS protocol incorporates the internal jugular vein (and where feasible femoral or subclavian waveforms) and can be combined with lung-ultrasound B-lines to capture pulmonary congestion not interrogated by the venous score [93,94]; these add-ons complement rather than replace the original grading and are less well characterized prognostically.

Several limitations qualify VExUS integration into standard protocols. Intrarenal venous Doppler is attenuated in advanced CKD, where parenchymal fibrosis alters waveform morphology independently of volume status [93]; right ventricular dysfunction, severe tricuspid regurgitation, and pulmonary hypertension can sustain abnormal hepatic and portal waveforms after volume correction, generating a false impression of persistent congestion. A dilated IVC carries adverse prognostic information independently of tricuspid regurgitation velocity, so combining standard echocardiographic parameters with the venous score helps differentiate genuine volume excess from isolated pulmonary hypertension or right ventricular disease [95]. Inter-operator reproducibility is substantial in trained hands (intraclass correlation coefficient ~0.83 for the overall grade) but requires structured training [96]. No randomized trial has yet demonstrated that VExUS-guided SNB improves mortality or rehospitalization; current use is best framed as reasoned bedside integration. Measurement at admission, at 48–72 h, and before discharge matches the time course of decongestion and avoids declaring resolution on peripheral signs alone [89,90,92].

### 6.9. Electrolyte Targets

Electrolyte targets, action thresholds and correction protocols during active sequential blockade are summarized in Table 3d. A creatinine rise of 10–20% from baseline during effective decongestion is expected, usually reflects hemoconcentration, and does not by itself warrant diuretic reduction; a fractional excretion of urea < 35% supports pre-renal physiology and argues against intrinsic tubular injury [5,21].

### 6.10. Ultrafiltration as a Salvage Option

Ultrafiltration is appropriate when (i) intravenous loop diuretics at optimal dose, combined with at least one adjunct from a different nephron segment, have failed to produce adequate decongestion; (ii) clinically significant congestion persists and the patient is hemodynamically stable enough to tolerate volume removal; and (iii) all reversible pharmacokinetic causes have been addressed. The CARRESS-HF trial, which used a fixed fluid removal rate of 200 mL/h in 188 patients with worsening cardiorenal syndrome, found that ultrafiltration was inferior to stepped pharmacological therapy on the bivariate primary endpoint of change in creatinine and weight at 96 h, with a mean creatinine rise of +20 µmol/L (+0.23 mg/dL) in the ultrafiltration arm versus −4 µmol/L (−0.04 mg/dL) with diuretics and no difference in weight loss. Serious adverse events also occurred more often with ultrafiltration [80]. The fixed 200 mL/h rate used in CARRESS-HF differs from current individualized approaches to fluid removal [97], although a standardized contemporary protocol has not been validated in a comparable randomized trial. Ultrafiltration cannot correct the underlying neurohormonal and tubular mechanisms driving diuretic resistance, and pharmacological combination therapy should be continued or reintroduced alongside mechanical fluid removal whenever feasible [6,79]. Serial bedside imaging during active fluid removal, combining echocardiographic stroke volume assessment with inferior vena cava diameter and pulmonary B-lines, can help identify impending over-decongestion before it manifests as a rise in creatinine or symptomatic hypotension [87].

## 7. Safety Considerations and a Focus on Older Patients

Electrolyte disturbances are the principal safety concern. Hypokalemia, the most common, results from increased distal sodium delivery to collecting duct ENaC, which drives potassium secretion proportionally. In CLOROTIC, it occurred significantly more often in the hydrochlorothiazide arm than with furosemide alone [69]. Thiazide additions amplify this risk. Mineralocorticoid antagonists added to loop-and-thiazide therapy partially offset potassium losses at the cost of hyperkalemia risk, particularly in CKD.

Hyponatremia is the principal serum sodium concern with thiazide-type augmentation. The mechanism combines impaired free-water excretion at the cortical diluting segment with stimulated thirst and arginine vasopressin release in patients with reduced effective arterial volume [12]. Risk concentrates in older patients; CLOROTIC enrolled at a median age of 83 years [69]. It is also higher in patients taking vasopressin-potentiating drugs such as selective serotonin reuptake inhibitors (SSRIs). Onset is typically within 1–4 weeks of initiation. In severe thiazide-induced hyponatremia (sodium < 125 mmol/L), withdrawal of the thiazide rather than fluid restriction alone is typically the appropriate clinical response [12]. Tolvaptan is reserved for documented dilutional hyponatremia (sodium < 130 mmol/L) where free-water excretion is the operative deficit; aquaresis without natriuresis offers no advantage in volume overload alone [50,51]. Hypomagnesemia accumulates from combined loop and thiazide therapy through paracellular thick ascending limb losses and downregulation of distal TRPM6 channels [13] and renders potassium replacement ineffective until corrected. Maintenance of serum magnesium at or above 0.8 mmol/L during active diuresis supports sustained potassium homeostasis and reduces the risk of arrhythmia in this setting [3].

Hypochloremic metabolic alkalosis from sustained loop-and-thiazide therapy contributes to diuretic resistance in its own right. Chloride depletion impairs tubuloglomerular feedback, may activate RAAS, and independently predicts worse outcomes in several heart failure cohorts [62,72]. Acetazolamide generates metabolic acidosis via bicarbonate excretion, restoring chloride balance during combination therapy. Whether this contributes to the efficacy observed in ADVOR beyond proximal sodium delivery is a mechanistic hypothesis; the trial was not designed to separate the two effects.

Hyperkalemia is the dose-limiting toxicity for potassium-sparing diuretics [98]. The risk of potassium elevation rises substantially at an eGFR below 30 mL/min/1.73 m^2^ when amiloride or triamterene is combined with ACE inhibitors, ARBs, ARNIs, or mineralocorticoid receptor antagonists. Intensified monitoring is warranted across the 30–60 mL/min/1.73 m^2^ range [35,36]. SGLT2i co-administration does not appear to add to this risk and may attenuate it, although the supporting analyses are post hoc and no trial was designed to test the interaction. Within the MRA class, finerenone in FINEARTS-HF approximately doubled the hazard of serum potassium exceeding 5.5 mmol/L versus placebo, with potassium above 6.0 mmol/L in 3.0% versus 1.4% [31,32]. The risk profile of steroidal MRAs is comparable. Combining more than one potassium-sparing agent with maximally tolerated RAAS blockade is generally avoided outside specialist supervision, in line with current ESC heart failure guidance [35]. Serum potassium and creatinine should be checked before initiation, within one week, at one month, and at every dose adjustment [35].Worsening renal function during decongestion is common, is often transient, and does not consistently predict adverse outcomes in patients without severe baseline CKD. Two situations have to be told apart at the bedside. In hemoconcentration, parallel rises in hematocrit and albumin accompany the creatinine increase and indicate effective volume removal. In true tubular injury, creatinine rises without hemoconcentration, alongside falling urine output and worsening acidosis. The two have opposite therapeutic implications [66].

Symptomatic hypotension and orthostatic intolerance limit the tolerability of layered combinations in elderly patients and in those with HFpEF and small, stiff ventricles, where preload reduction is poorly tolerated. In EMPULSE, volume depletion adverse events occurred in 12.7% versus 10.2% of patients, while serious symptomatic hypotension was uncommon and balanced between arms (1.2% versus 1.5%) [43]; SOLOIST-WHF reported hypotension in 6.0% versus 4.6% [47]. The risk of orthostatic intolerance rises with concurrent ARNI and SGLT2i exposure. Both supine and standing blood pressure readings are informative in patients on multiple agents with combined negative inotropic, vasodilatory or natriuretic effects. Euglycemic ketoacidosis with SGLT2i is uncommon in heart failure cohorts (2/605 vs. 4/611 in SOLOIST-WHF [47]; comparably low rates in EMPULSE [43]).

In line with prescribing-information guidance for empagliflozin and dapagliflozin, temporary discontinuation is recommended during prolonged fasting, intercurrent illness, or perioperative care. The risk is most relevant in patients with type 1 diabetes or insulin-dependent type 2 diabetes.

A further question arises now that SGLT2i therapy is usually already in place at the moment of decompensation, a situation almost absent from the trials that inform this review (Section 8). SGLT2 inhibition raises distal sodium delivery to the macula densa and restores tubuloglomerular feedback, which accounts for the early fall in the eGFR seen after initiation. Adding acetazolamide to such a patient layers a second proximally acting agent onto the same mechanism, so part of the natriuretic effect may be redundant while the acid load is not; adding a thiazide acts on a distal convoluted tubule that may already be adapted to a higher sodium load. Neither combination has been examined in a randomized trial, and the network meta-analytical data discussed in Section 8 show a real, if imprecisely estimated, renal signal for all three augmentation strategies. Pending direct evidence, a defensible approach in the patient already stabilized on an SGLT2i is to start the adjunct at the lower end of its dose range, to measure sodium, potassium, chloride, bicarbonate and creatinine at 12 and 24 h rather than at 24 h alone, and to interpret an early creatinine rise against hematocrit and albumin before attributing it to tubular injury. Interrupting the SGLT2i is not warranted for decongestion alone and should be reserved for the circumstances noted above. Older patients with frailty warrant particular caution, and the hazards of aggressive decongestion in this group extend across several domains that the trial populations represent only in part. Rapid volume removal together with thiazide- or tolvaptan-related shifts in serum sodium can precipitate delirium, while orthostatic hypotension from layered natriuretic and vasodilator therapy raises the probability of falls and injury. Sarcopenia, blunted thirst, and limited independent access to fluids amplify these risks, so a decongestive target appropriate for a younger patient may produce symptomatic hypovolemia in an older one. Lower starting doses of any adjunct, closer electrolyte surveillance and earlier de-escalation are therefore prudent. Medication reconciliation should be completed before discharge and a structured review of weight, blood pressure, creatinine, sodium, potassium, magnesium and chloride arranged within 7 to 14 days. Across this trajectory, the therapeutic goal shifts from fluid removal alone to the preservation of function, mobility, and safety. In this context, the geriatric vulnerability phenotype summarized in Table 4 intersects with the biochemical phenotypes rather than representing a distinct category.

## 8. Limitations of Current Evidence

The randomized evidence informing sequential nephron blockade has methodological limitations that should be considered before its findings are systematized into a fixed protocol. No augmentation strategy has been compared against another in adequately powered head-to-head trials. Available comparators are placebo (CLOROTIC, ADVOR, EMPULSE, PUSH-AHF) or active controls chosen for pragmatic rather than mechanistic reasons (DAPA-RESIST against metolazone). DEA-HF compared three regimens within patients in a 42-patient crossover, and the 3T trial offered an underpowered three-way comparison among metolazone, intravenous chlorothiazide, and tolvaptan in 60 patients with no design power for non-inferiority. The relative efficacy of thiazide augmentation versus acetazolamide addition in patients with documented distal braking, and whether SGLT2i initiation matches either at equivalent background loop dose, is presently conjectural. A recent systematic review of twelve trials reached a similar conclusion, documenting consistent short-term decongestion gains across augmentation strategies alongside transient creatinine elevation and the absence of an established mortality or rehospitalization benefit for any single combination [99].

Two network meta-analyses have since attempted the indirect comparison that no head-to-head trial provides. The first, covering 25 randomized trials and 7149 patients admitted for acute heart failure irrespective of documented resistance, found that continuous furosemide infusion and the addition of tolvaptan, of a thiazide or of an SGLT2i each produced greater 24 h weight loss than furosemide boluses alone, with odds ratios of 1.55 (95% confidence interval 1.39–1.63), 1.57 (1.39–1.77), 1.63 (1.37–1.94) and 1.23 (1.06–1.42), respectively. Worsening renal function was more frequent with acetazolamide (1.81, 1.31–2.49), with a thiazide (1.78, 1.43–2.21) and with an SGLT2i (1.52, 1.19–1.94), and hypokalemia was more frequent with a thiazide (1.69, 1.32–2.16). The pattern for acetazolamide deserves particular attention: it was the only augmentation strategy that did not increase weight loss (1.01, 0.78–1.32) while carrying the highest odds of worsening renal function. An underpowered exploratory analysis showed a non-significant reduction in mortality or rehospitalization with an SGLT2i (0.45, 0.19–1.07) [100]. The second analysis, restricted to trials enrolling patients with established diuretic resistance, reached a compatible conclusion: add-on strategies increased short-term urine output and weight loss with imprecise estimates and a higher risk of acute kidney injury, whereas an SGLT2i was the only strategy associated with fewer heart failure hospitalizations and with a renal safety profile that did not deteriorate [101].

Neither analysis substitutes for direct comparison. In both networks each strategy is compared against loop diuretic boluses rather than against the other strategies, so the comparison that matters clinically, thiazide against acetazolamide against an SGLT2i, is derived indirectly and rests on a transitivity assumption that the constituent trials do not satisfy: background loop diuretic dose, timing of randomization relative to admission, eGFR eligibility and the era of SGLT2i use differ systematically between the acetazolamide, thiazide and SGLT2i arms. Statistical heterogeneity was moderate to high across endpoints, worsening renal function was defined differently between trials, and the outcomes carrying the greatest clinical weight were reported in roughly half of the studies. What both analyses support is therefore a negative statement rather than a ranking: no augmentation strategy has shown superiority over another, and each buys short-term decongestion at some renal or electrolyte cost.

Endpoint heterogeneity confounds cross-trial inference. Weight loss at 72 or 96 h, the dominant primary endpoint in CLOROTIC and DAPA-RESIST, captures gross fluid removal but does not adjudicate residual neurohormonal activation, which may persist after weight stabilization. Twenty-four-hour natriuresis, one of the two co-primary endpoints in PUSH-AHF, reflects diuretic efficacy directly and was met by the natriuresis-guided strategy; the co-primary 180-day composite of death and HF rehospitalization was not. The hierarchical clinical-benefit composite of EMPULSE combines death, heart failure events and symptom change into a single win-ratio estimate; the result is method-specific and cannot be compared directly with traditional time-to-event analyses. Worsening renal function during decongestion has been used as a safety endpoint across all major trials, although, as discussed in Section 7, evidence from large heart failure cohorts indicates that aggressive-diuresis-related creatinine elevation is uncoupled from biomarkers of tubular injury and does not consistently predict adverse outcomes [83,84].

Patients with advanced chronic kidney disease are systematically underrepresented. ADVOR included eGFRs down to approximately 20 mL/min/1.73 m^2^ but enrolled few with established stage 4 disease. CLOROTIC stratified hydrochlorothiazide dose by the eGFR but excluded eGFRs below 15 mL/min/1.73 m^2^; the post hoc eGFR analysis (detailed in Section 4.2) documented attenuating weight-loss benefit at lower function, with the strongest signal lost in patients with an eGFR < 45. EMPULSE allowed eGFRs down to 20 mL/min/1.73 m^2^ but enrolled few patients near the lower bound, FINEARTS-HF excluded eGFRs < 25, and the median eGFR in SOLOIST-WHF was approximately 50 mL/min/1.73 m^2^. Recent KDIGO consensus on kidney disease and heart failure has acknowledged that the cardiorenal patient most in need of treatment guidance is the one least represented in randomized cohorts [86]. The efficacy of acetazolamide at an eGFR of 15–25 is unknown. Finerenone safety with concurrent RAAS blockade in stage 4 disease has not been formally established [32], and SGLT2i tolerability below current inclusion thresholds (dapagliflozin < 25, empagliflozin < 20) rests on extrapolation rather than direct evidence.

Major trials were conducted before the widespread adoption of SGLT2is as guideline-directed therapy, a gap of relevance to contemporary practice. ADVOR, CLOROTIC, DOSE, and CARRESS-HF were either conducted before SGLT2i uptake or specifically excluded patients receiving them. Background SGLT2i use was approximately 14% in FINEARTS-HF, was an explicit exclusion at randomization in ADVOR, and was negligible in CLOROTIC given enrollment dates. The natriuretic effects of acetazolamide, hydrochlorothiazide or amiloride in patients already receiving an SGLT2i at the moment of decompensation remain untested in adequately powered randomized settings. SGLT2 inhibition acts in part on the proximal tubule. Acetazolamide’s contribution may therefore be partly redundant in SGLT2-treated patients, while distal blockade with thiazides or amiloride may act on a more upregulated DCT than in the historical trial populations on which current evidence rests. Both statements are mechanistic predictions and neither has been tested experimentally.

## 9. Conclusions

Inadequate natriuretic response to intravenous furosemide in acute decompensated heart failure typically reflects several converging mechanisms. WNK–SPAK-mediated NCC upregulation usually coexists with aldosterone-driven distal sodium avidity; in advanced congestion, saturation of the glycosaminoglycan-bound interstitial sodium pool can further blunt the clinical response despite documented natriuresis. In patients with concurrent CKD, tubular delivery of loop diuretics is additionally constrained by competitive inhibition of OAT-mediated secretion. Whether biomarkers will enter future escalation algorithms depends on which of them can be tied to an action rather than to a prognosis. Spot urinary sodium is the only candidate with randomized support for guiding treatment, and PUSH-AHF delivered what it was designed to deliver: greater natriuresis, without benefit on the 180-day clinical composite. Serum and urinary chloride are attractive complements because chloride sits upstream of the transporters being blocked, but no trial has randomized patients on a chloride threshold. Among circulating markers, carbohydrate antigen 125 has the most mature outcome data, from a randomized trial in which a guided strategy after discharge reduced death or readmission at one year [102,103]; that trial guided post-discharge intensity rather than in-hospital escalation, so its result does not transfer to the decision addressed here. Venous congestion imaging remains at an earlier stage [92], while tubular injury markers occupy a different place in the algorithm, arguing against reflexive de-escalation when creatinine rises during effective diuresis rather than triggering escalation [83,84]. A composite trigger combining natriuresis at 2 h, serum chloride and a venous congestion score is the plausible next step, but it will be worth adopting only if tested prospectively with action thresholds fixed in advance.

For the clinician at the bedside, the practical content of this review reduces to a short sequence: confirm that loop diuretic dose and route are adequate before concluding that resistance exists, measure spot urinary sodium at 2 h, and escalate the loop dose once. If natriuresis remains below threshold, stop escalating and choose the adjunct that matches the biochemical pattern rather than the one that is habitual on the ward, correcting potassium, magnesium and chloride at the same time. Add one agent at a time, measure the response before adding another, and treat an isolated creatinine rise in a patient who is decongesting as expected rather than as a reason to stop. Reassess within two weeks of discharge, since the electrolyte consequences of combination therapy usually declare themselves after the patient has left. What would change this sequence is direct comparative evidence: a head-to-head comparison of a thiazide against acetazolamide in patients with documented distal escape, inclusion rather than exclusion of eGFRs below 30 mL/min/1.73 m^2^, randomization on a background of established SGLT2i therapy, and endpoints harmonized around natriuresis at 6 and 24 h together with a clinical composite.

## 10. Key Points

- Inadequate natriuretic response to intravenous loop diuretics arises from several coexisting mechanisms, including impaired tubular drug delivery, WNK-SPAK-mediated distal sodium cotransporter upregulation, neurohormonal sodium avidity, and post-diuretic sodium retention; escalation of the loop dose alone addresses only a subset of these.
- Sequential nephron blockade is most rational when the adjunct agent is matched to the predominant mechanism of sodium retention, moving the approach from empirical combination therapy toward mechanism-based treatment.
- Randomized evidence of heterogeneous quality supports thiazide augmentation (CLOROTIC), acetazolamide addition (ADVOR), in-hospital SGLT2 inhibitor initiation (EMPULSE), and natriuresis-guided escalation (PUSH-AHF), while adequately powered head-to-head comparisons of these strategies are lacking.
- Early spot urinary sodium measurement and biochemical phenotyping, supported where available by venous congestion ultrasound, allow individualized escalation and de-escalation of combination diuretic therapy.
- Patients with advanced chronic kidney disease and those already established on SGLT2 inhibitors remain underrepresented in the trial evidence, leaving mechanism-matched therapy in these groups an open question.

## Figures and Tables

**Table 1:** Pharmacological properties of diuretic agents used in sequential nephron blockade.

Agent	Class/Nephron Site	Primary Target	Oral Bioav. (%)	t½ (h)	Elimination	Key Clinical Notes
Furosemide	Loop/TALH	NKCC2	10–90	0.5–2 (healthy); ~10 (range up to 24) in advanced CKD	~50% renal unchanged; UGT1A9/1A1 glucuronidation (renal + hepatic)	High variability; t½ ↑ in CKD; IV bypasses gut edema
Torsemide	Loop/TALH	NKCC2	80–90	3–4	Hepatic (CYP2C9)	Less PK variability than furosemide (hepatic CYP2C9); TRANSFORM-HF: no mortality difference
Bumetanide	Loop/TALH	NKCC2	80–95	1–1.5	~80% renal (about half as unchanged drug, half as hepatic side-chain oxidation metabolites); biliary ~2%	More consistent absorption than furosemide; 40 mg furosemide ≈ 1 mg bumetanide; shorter t½ favors bid/tid dosing
Ethacrynic acid	Loop/TALH	NKCC2	~100	<1 (mean ~0.5)	Biliary + renal	Non-sulfonamide; reserved for sulfonamide allergy; significant ototoxicity
Hydrochlorothiazide	Thiazide/DCT	NCC	60–80	6–15	Renal	Effect attenuates as eGFR falls (CLOROTIC post hoc: minimal benefit at eGFR < 45); metolazone or chlorthalidone preferred at eGFR < 30
Chlorothiazide IV	Thiazide/DCT	NCC	N/A (IV)	~2	Renal	Rapid tubular peak; no pre-dosing needed; costly; use when oral absorption compromised
Chlorthalidone	Thiazide-like/DCT	NCC	65–75	40–60	Renal	High RBC affinity (Vd 5–10 × HCTZ); active to eGFR 20; preferred for outpatient SNB
Metolazone	Thiazide-like/DCT (PT effect proposed)	NCC (additional proximal effect proposed in older work)	50–60	8–14	Renal	Variable absorption in gut edema; activity reported in advanced CKD (observational); administer 30–60 min pre-loop
Indapamide	Thiazide-like/DCT	NCC + vasodilation (Ca channel; prostaglandin)	~93	14–18	Hepatic	No renal dose adjustment; dual vasodilatory effect; limited SNB trial data
Spironolactone	MRA/CCD	MR (non-selective)	60–90	~1.4 (parent); 14–24 (canrenone)	Hepatic (active metabolites)	Effect sustained 24–48 h via canrenone; renal accumulation > cardiac; RALES [18]; gynecomastia ~10–15%
Potassium canrenoate (→canrenone)	MRA (steroidal)/CCD	MR (non-selective)	N/A (IV)	~13.5–24 (without HF or hepatic dysfunction); 37 in CHF, 59 in hepatic failure	Hepatic (active canrenone); renal excretion of metabolites	Only parenteral MRA; licensed in several European countries (not USA); 100–200 mg IV od–bid; tolerability of higher doses reported in a single cohort [23]; prospective efficacy data absent
Eplerenone	MRA/CCD	MR (selective)	~69	4–6	Hepatic (CYP3A4)	Greater MR selectivity than spironolactone; reduced sex-hormone adverse effects; EMPHASIS-HF [28]
Finerenone	Non-steroidal MRA/CCD	MR (high affinity)	~43	2–3	Hepatic (CYP3A4)	Equal heart/kidney distribution; hyperkalemia remains a class effect; FINEARTS-HF [32] (HFpEF/HFmrEF); no active metabolites
Amiloride	ENaC blocker/CCD	ENaC (direct)	~50	6–9	Renal (unchanged)	No hepatic metabolism; Mg conservation; t½ ↑ in CKD; useful in nephrotic ENaC activation
Triamterene	ENaC blocker/CCD	ENaC (direct)	30–70	1.5–2.5 parent; duration of action ~12–16 h via active metabolite	Hepatic (active metabolite, renal excretion)	Mg excretion ↑ (unlike amiloride); crystalline nephropathy; bioavailability increased with high-fat meal
Acetazolamide	CAI/PCT	Carbonic anhydrase II	~90	6–9	Renal (unchanged)	ADVOR: 500 mg IV × 3 d; can mitigate hypochloremic alkalosis; DEA-HF (small, ambulatory, 95% on SGLT2i) did not show incremental benefit
Empagliflozin	SGLT2i/PCT	SGLT2	~75	12–17	UGT glucuronidation	EMPULSE; no CYP interactions; limit eGFR 20; decongestion via TGF/RAAS modulation, not direct natriuresis
Dapagliflozin	SGLT2i/PCT	SGLT2	~78	12–17	UGT glucuronidation	DAPA-RESIST: comparable decongestion to metolazone in loop-resistant HF (not a non-inferiority trial); fewer electrolyte disturbances; limit eGFR 25
Sotagliflozin	SGLT1/2i/PCT + gut	SGLT2 + SGLT1	~25 absolute; enterohepatic circulation contributes ~50% of systemic exposure	21–35	UGT	Dual renal + intestinal mechanism; SOLOIST-WHF: HR 0.67 in worsening HF; US approval (2023) for HF across full LVEF range and for T2D + CKD + CV risk factors; not yet approved in the European Union for HF; not marketed for glycemic control
Tolvaptan	V2 antagonist/CCD	V2 receptor (AVP)	~56	~12	Hepatic (CYP3A4)	Aquaresis (water only, no Na+); EVEREST: no survival benefit; role limited to dilutional hyponatremia (Na < 130) with congestion

Pharmacokinetic and pharmacodynamic properties of agents used in sequential nephron blockade. Bioavailability values are mean estimates from published pharmacokinetic studies; half-life ranges reflect variability across eGFR strata where applicable. Clinical notes summarize key context for use in acute decompensated heart failure. ↑, increased; AVP: arginine vasopressin; CAI: carbonic anhydrase inhibitor; CCD: cortical collecting duct; CKD: chronic kidney disease; CYP2C9: cytochrome P450 2C9; CYP3A4: cytochrome P450 3A4; DCT: distal convoluted tubule; eGFR: estimated glomerular filtration rate; ENaC: epithelial sodium channel; HF: heart failure; HFpEF: heart failure with preserved ejection fraction; IV: intravenous; MR: mineralocorticoid receptor; MRA: mineralocorticoid receptor antagonist; NCC: Na-Cl cotransporter; NKCC2: Na-K-2Cl cotransporter 2; PCT: proximal convoluted tubule; PK: pharmacokinetics; PT: proximal tubule; RAAS: renin–angiotensin–aldosterone system; RBC: red blood cell; SGLT1: sodium–glucose cotransporter 1; SGLT2: sodium–glucose cotransporter 2; SNB: sequential nephron blockade; TALH: thick ascending limb of Henle; TGF: tubuloglomerular feedback; UGT: UDP-glucuronosyltransferase; V2: vasopressin V2 receptor; Vd: volume of distribution.

**Table 2:** Key randomized trials informing sequential nephron blockade strategies in acute heart failure.

Trial	N	Population	Intervention	Primary Result	Year [Ref]
DOSE	308	ADHF on chronic oral loop diuretic (80–240 mg/day furosemide-equivalent)	High vs. low IV furosemide dose; bolus vs. infusion	High dose: greater diuresis and symptom improvement; transient ↑ Cr; no mortality difference; continuous vs. bolus: no significant difference	2011 [75]
TRANSFORM-HF	2859	Hospitalized HF, all phenotypes	Torsemide vs. furosemide (discharge strategy)	No difference in all-cause mortality (26.1% vs. 26.2%; HR 1.02); no difference in HF hospitalization or QoL at 12 months	2023 [9,10]
CLOROTIC	230	ADHF, IV furosemide background	Hydrochlorothiazide 25–100 mg (eGFR adjusted: >50 → 25 mg; 20–50 → 50 mg; <20 → 100 mg) + furosemide IV vs. furosemide + placebo	Hydrochlorothiazide: greater weight loss at 72 h (−2.3 vs. −1.5 kg; adjusted difference 1.14 kg (95% CI 0.42–1.84); *p* = 0.002); secondary endpoint at 96 h (−2.5 vs. −1.5 kg; *p* < 0.001); benefit consistent across HFrEF/HFmrEF/HFpEF (CLOROTIC-EF sub-analysis)	2023 [69,70]
3T Trial	60	ADHF with loop diuretic resistance	Metolazone vs. IV chlorothiazide vs. tolvaptan + high-dose furosemide	No significant between-group difference in 48 h weight loss or cumulative urine output; not designed as a non-inferiority trial	2020 [52]
ADVOR	519	ADHF, elevated NP, IV loop diuretic background (median oral dose 60 mg/day)	Acetazolamide 500 mg IV once daily × 3 days vs. placebo	Successful decongestion at day 3: 42.2% vs. 30.5% (RR 1.46; 95% CI 1.17–1.82); no significant ↑ WRF or hypokalemia	2022 [39]
DAPA-RESIST	61	ADHF with documented loop diuretic resistance	Dapagliflozin 10 mg/d vs. metolazone 5–10 mg/d × 4 days	Weight change at 96 h: −3.0 vs. −3.6 kg (difference 0.65 kg, 95% CI −0.12 to 1.41; *p* = 0.11); dapagliflozin: significantly fewer hypokalemia and hyponatremia events (not a formal non-inferiority design)	2023 [44]
EMPULSE	530	Hospitalized AHF (all EF; 45% with type 2 diabetes)	Empagliflozin 10 mg initiated in-hospital after clinical stabilization (median 3 days) vs. placebo	Clinical benefit (hierarchical composite): win ratio 1.36 (95% CI 1.09–1.68) at 90 days; safe early initiation; improved diuretic efficiency	2022 [43,74]
SOLOIST-WHF	1222	T2DM + worsening HF (post-discharge initiation)	Sotagliflozin vs. placebo (initiated before or within 3 days of discharge)	Total (first and recurrent) CV death, HF hospitalization, urgent HF visits: 51.0 vs. 76.3 events per 100 patient-years (HR 0.67, 95% CI 0.52–0.85; *p* < 0.001); trial terminated early after sponsor withdrawal; primary endpoint revised to total-event composite [47]	2021 [47]
PUSH-AHF	310	AHF requiring IV loop diuretics	Natriuresis-guided therapy (UNa trigger < 70 mmol/L) vs. standard care	24 h natriuresis: 409 vs. 345 mmol (adjusted between-group difference 63 mmol, 95% CI 18–109; *p* = 0.006); all-cause mortality or HF rehospitalization at 180 days: 31% vs. 31% (HR 0.92, 95% CI 0.62–1.38; *p* = 0.70); numerical signal in low-eGFR subgroup (hypothesis-generating); post hoc BAN-ADHF analysis [76] showed consistent natriuretic benefit across diuretic-resistance strata	2023 [77,78]
CARRESS-HF	188	Worsening cardiorenal syndrome after ADHF	Ultrafiltration (fixed 200 mL/h) vs. stepped pharmacological care	UF inferior to pharmacological care on bivariate endpoint (ΔCr +20 vs. −4 µmol/L (+0.23 vs. −0.04 mg/dL); *p* = 0.003; weight change not different); serious adverse events higher with UF (72% vs. 57%, *p* = 0.03)	2012 [79]
PROTECT	2033	Hospitalized AHF with renal dysfunction	Rolofylline 30 mg IV/day × 3 days vs. placebo	No benefit on primary trichotomous composite endpoint (OR 0.92, 95% CI 0.78–1.09; *p* = 0.35); persistent renal impairment in 15.0% vs. 13.7% (*p* = 0.44); seizures more frequent in the rolofylline arm (11/1356, 0.8%, vs. 0/677; *p* = 0.02; central A1 class effect)	2010 [53]

Characteristics and primary results of key randomized controlled trials providing the evidence base for sequential nephron blockade strategies in acute heart failure and diuretic resistance. Trial design, sample size, intervention, primary endpoint, and key findings are summarized for each study. Outcomes are reported as originally published; non-inferiority or superiority status reflects the prespecified trial hypothesis. Trials are grouped by intervention class. ↑, increased; ADHF: acute decompensated heart failure; AHF: acute heart failure; CI: confidence interval; Cr: creatinine; CV: cardiovascular; eGFR: estimated glomerular filtration rate; HF: heart failure; HFmrEF: heart failure with mildly reduced ejection fraction; HFpEF: heart failure with preserved ejection fraction; HFrEF: heart failure with reduced ejection fraction; HR: hazard ratio; IV: intravenous; NP: natriuretic peptides; OR: odds ratio; RR: relative risk; T2DM: type 2 diabetes mellitus; UF: ultrafiltration; UNa: urinary sodium concentration; WRF: worsening renal function.

**Table 3:** (**a**). Natriuresis-guided escalation: decision thresholds and timepoints. (**b**). Hypothesized phenotype–adjunct associations in loop-diuretic-resistant ADHF. (**c**). Diuretic agent selection stratified by eGFR. (**d**). Electrolyte management during active sequential nephron blockade.

**(a). Natriuresis-Guided Escalation: Decision Thresholds and Timepoints**					
**Time point**	**Parameter**	**Adequate** **Response**	**Action If Inadequate**	**Caveats**	
Baseline (pre-dose)	Spot urinary Na (baseline); BMP; Mg; creatinine; urea; body weight (calibrated scale, after void, comparable clothing)	Establishes reference; no threshold applicable	Set IV dose at approximately ×2 the outpatient oral daily dose (≈×2.5 if CKD or known gut edema, the multiplier used in the high-dose arm of the DOSE trial [75]; the ×2 baseline reflects common clinical practice rather than a DOSE-derived value). Administer as IV bolus, preferably twice daily; once-daily dosing leaves 16–21 h of post-diuretic Na retention unchecked [3,21].	Record pre-dose UNa: without it the 2 h comparison is uninterpretable. Consider switching to bumetanide or torsemide in outpatients with documented gut edema (more predictable bioavailability).	
2 h post-dose	Spot urinary Na	≥70 mmol/L: adequate response; continue and recheck at 6 h	<70 mmol/L (PUSH-AHF trigger [77]): double the loop diuretic dose and recheck 2 h UNa after the next bolus. After two consecutive failed doublings, add an adjunct from a different nephron segment (see Table 3b). A more aggressive <50 mmol/L threshold has been proposed [3,14] and may be applied at centers preferring earlier escalation.	AKI/ATN: UNa high despite oliguria; do not confuse with adequate natriuresis. SGLT2i: glucose-driven osmotic diuresis dilutes UNa; interpret with UO and urine glucose. Severe hypoalbuminemia (<20 g/L): impaired OAT secretion; consider IV albumin co-infusion (limited RCT evidence).	
6 h post-dose	Urine output	>100–150 mL/h [21]; widely used clinical threshold	If not achieved despite dose doubling: identify biochemical phenotype (Table 3b) and add adjunct from a different nephron segment.	After two consecutive failed loop doublings (per Felker et al. [3]), further loop escalation has low yield; consider combination from a different nephron segment	
24 h post-dose	Weight loss; diuretic efficiency (kg lost per 40 mg furosemide equivalent)	0.5–1.5 kg/24 h (commonly applied clinical target); 1–2 kg/24 h (moderate congestion); 2–3 kg/24 h (severe congestion, if systolic BP tolerates)	Low diuretic efficiency: suspect pharmacokinetic barrier, dietary sodium intake, or third-compartment sodium buffering.	Hemoconcentration (rising Hct + albumin + Cr) is the desired direction. Isolated Cr rise with falling UO and worsening acidosis = probable ATN: reduce loop diuretic, do not escalate.	
Q12h (first 48 h on combination therapy)	Na, K, Cl, HCO_3_, Cr, Mg	K ≥ 3.5 mmol/L; Mg ≥ 0.8 mmol/L; Cl ≥ 96 mmol/L; HCO_3_ 22–28 mmol/L	See Table 3c for electrolyte correction thresholds and infusion rates.	Switch to every 24 h after first 48 h once electrolytes stable.	
**(b). Hypothesized Phenotype–Adjunct Associations in Loop-Diuretic-Resistant ADHF.**					
**Biochemical Phenotype**	**Pattern**	**Preferred Adjunct**	**Dose and Timing**	**Cautions**	
Braking phenomenon (NCC upregulation)	Hypokalemia; adequate UNa response initially but rebound sodium retention; prior chronic loop use (>3–6 months); declining 24 h diuretic efficiency over successive days	Metolazone (1st choice; CKD-tolerant in observational data); HCTZ (eGFR > 30); chlorthalidone (outpatient SNB, eGFR > 20)	Metolazone 2.5–5 mg PO (up to 10 mg); 30–60 min pre-loop bolus; HCTZ 25–50 mg PO; chlorthalidone 12.5–25 mg PO od	Evidence: RCT (CLOROTIC [69]) for HCTZ add-on, observational for metolazone CKD tolerance. Prefer metolazone or chlorthalidone at eGFR < 30 (greater retained activity at low GFR); HCTZ at the CLOROTIC dose for eGFR < 20 (100 mg/day) is a defensible alternative when these are unavailable, with intensified monitoring of serum sodium, potassium and creatinine. Correct K and Cl before or simultaneously: NCC blockade with electrolyte depletion worsens WNK activation.	
Hypochloremic alkalosis (WNK activation by Cl depletion)	Cl < 96 mmol/L; HCO_3_ > 30 mmol/L; poor natriuresis disproportionate to UNa concentration; hyponatremia (Na < 135) or eunatremia	HSS (3% NaCl 100 mL) if Na < 135, co-infused with furosemide KCl IV if Na normal and K low; acetazolamide can mitigate alkalosis and help restore Cl balance	HSS (investigational, center-specific): 100 mL 3% NaCl over 30–60 min before or with furosemide bolus; KCl: 10–20 mmol/h IV peripheral (max 40 mmol/L concentration)	Evidence: post hoc subgroup in SALT-HF (ambulatory worsening HF, not ADHF; *n* = 16 hypochloremic patients; see Section 6.6) [82]. HSS co-infusion is investigational and center-specific; prospective confirmation required before routine use. Do not add thiazide if Na < 135; worsens hyponatremia via vasopressin potentiation.	
Proximal sodium avidity (neurohormonal; low FENa)	Pre-dose UNa < 20 mmol/L; hypochloremic alkalosis; relatively low chronic loop burden (oral dose ≤ 80 mg FE/day); elevated BUN/Cr ratio; HFrEF with high RAAS activation	Acetazolamide (increases proximal Na delivery to loop; counteracts alkalosis by bicarbonate excretion)	Acetazolamide 500 mg IV od × 3 days on standardized IV loop background (ADVOR regimen)	Evidence: RCT (ADVOR [39], N = 519) for acetazolamide 500 mg IV od × 3 days on intravenous loop background; decongestion endpoint, no significant effect on 3-month mortality or HF rehospitalization. ADVOR explicitly excluded patients on SGLT2i at randomization, and severe CKD was underrepresented, limiting extrapolation to contemporary practice. DEA-HF [41] (small, ambulatory, nearly all on SGLT2i) found no incremental benefit but is too limited in size and setting to resolve whether mechanistic overlap with SGLT2i negates the ADVOR effect. Benefit was most apparent at baseline oral loop ≤ 80 mg FE/day (ADVOR median 60 mg/day). ADVOR applied a fixed 500 mg IV od dose across the whole enrolled eGFR range and excluded patients below eGFR 20; a reduction to 250 mg IV od at eGFR < 40 (down to ~20) is a pragmatic extrapolation to limit additive acidosis rather than a trial-specified adjustment, and below eGFR 20 acetazolamide is best avoided given the absence of trial-derived data.	
Secondary hyperaldosteronism (aldosterone-driven ENaC upregulation)	Refractory hypokalemia despite replacement; eGFR > 30; RAAS activation (HFrEF, cirrhosis, nephrotic); limited DCT hypertrophy pattern	Spironolactone or eplerenone (eGFR ≥ 30, K ≤ 5.0 mmol/L); finerenone if concurrent DKD with albuminuria	Spironolactone 25–50 mg PO od; eplerenone 25–50 mg PO od; finerenone 20 mg PO od (eGFR 25 to <60 mL/min/1.73 m^2^, titrate from 10 mg); or 40 mg PO od (eGFR ≥ 60, titrate from 20 mg) per FINEARTS-HF strategy; potassium canrenoate 100–200 mg IV od–bid if oral route not feasible (where licensed); escalate to 300–400 mg/day in refractory cases under close biochemical surveillance [23]	Evidence: RCT (RALES [18], EMPHASIS-HF [28] for spironolactone/eplerenone in chronic HFrEF; FINEARTS-HF [32] for finerenone in HFpEF/HFmrEF). MRAs are principally outcome-modifying and potassium-sparing therapies rather than acute decongestive agents; their inclusion here addresses refractory hypokalemia and RAAS-driven distal sodium avidity in this subset, not acute natriuresis comparable to thiazide-type augmentation. Check K before each dose; hold if K > 5.5. Steroidal MRA: ESC 2021 thresholds for initiation in HF are eGFR ≥ 30 mL/min/1.73 m^2^ and K ≤ 5.0 mmol/L [35]. Natriuretic potency is modest; decongestive intent remains with loop, thiazide, and acetazolamide.	
Dilutional hyponatremia with congestion (ADH excess)	Na < 130 mmol/L; persistent edema; concentrated urine (osmolality > 300 mOsm/kg); low Cl; inadequate free water excretion	Tolvaptan (aquaresis; corrects the water retention component; does not address primary sodium avidity)	Tolvaptan 15 mg PO od; titrate to 30–60 mg based on daily Na response	Evidence: RCT (EVEREST) neutral for survival in unselected HF; tolvaptan use in dilutional hyponatremia with congestion is based on physiological rationale and label indication for hypervolemic hyponatremia rather than HF mortality data. Correct Na at ≤8–10 mmol/L/day (lower ceiling of 8 mmol/L/24h in patients at higher risk of osmotic demyelination syndrome). Do not combine with fluid restriction initially. Do not co-administer thiazides (exacerbates hyponatremia).	
Nephrotic syndrome (serine-protease ENaC activation)	Heavy proteinuria; loop diuretic resistance disproportionate to eGFR; poor natriuretic response despite IV route; low albumin	Amiloride (direct ENaC blockade overrides serine-protease cleavage activation) +/− IV albumin + furosemide if albumin < 20 g/L	Amiloride 5–10 mg PO od (reduce to 2.5–5 mg if eGFR < 45); albumin 100 mL 20% + furosemide 40–80 mg IV (selected patients with severe hypoalbuminemia)	Evidence: physiological inference and small observational series. Amiloride in nephrotic syndrome with reduced eGFR is off-label and rests on the mechanistic rationale for direct ENaC blockade in protease-driven sodium retention; confine to specialist settings with intensified potassium and creatinine monitoring. Albumin–furosemide co-infusion is not routinely recommended; randomized trial data are limited and inconsistent, and if used it should be reserved as a rescue option in severe hypoalbuminemia (<20 g/L) unresponsive to standard intensification. Amiloride preferred over triamterene in Mg-depleted states.	
**(c). Diuretic Agent Selection Stratified by eGFR**					
**Agent/Class**	**eGFR ≥ 60**	**eGFR 30–59**	**eGFR 15–29**	**eGFR < 15**	**Notes on CKD Dosing**
Loop diuretics (furosemide)	Standard IV dosing; oral:IV 2:1	Dose × 1.5–2.5; IV preferred; OAT competition ↑	Doses ≥ 200 mg IV bd common; consider torsemide/bumetanide PO for outpatient	Highest doses; diminishing returns from OAT competition; always IV	DOSE trial [75] high-dose arm used ×2.5 the outpatient oral dose IV; this ratio is clinically appropriate in low eGFR or high UACR where OAT competition reduces tubular drug delivery. Higher doses required because less drug reaches tubular lumen, not because per-molecule efficacy falls.
Hydrochlorothiazide	Yes; 25–50 mg PO	Attenuated below eGFR 45	No demonstrable weight-loss benefit in CLOROTIC post hoc at eGFR < 45 (point estimate −0.1 kg, CI crossed zero); 100 mg/day defensible per CLOROTIC stage 4 protocol with intensified monitoring; metolazone or chlorthalidone preferred	Avoid	Prefer chlorthalidone or metolazone in CKD G3b or worse.
Metolazone	Yes; 2.5–5 mg PO	Yes; preferred thiazide-like in CKD	Observational data support activity; possible proximal tubular effect	Case series only; monitor electrolytes intensively	Variable absorption in gut edema; administer 30–60 min pre-loop. Prolonged t½ (8–14 h); may accumulate with repeated dosing.
Chlorthalidone	Yes; 12.5–25 mg od	Yes; sustained NCC blockade (t½ 40–60 h); preferred for outpatient SNB	Activity to eGFR ~20; preferred over HCTZ in CKD G4	Limited data; use with caution	High RBC affinity; no concentration peaks; ideal once-daily agent for sustained DCT blockade.
Acetazolamide	Yes; 500 mg IV od (ADVOR)	500 mg IV od (eGFR 40–59); 250 mg IV od (eGFR 30–39); monitor closely	250 mg IV od (eGFR 20–29) with caution; below eGFR 20 generally avoided (outside ADVOR range); uremic acidosis risk; renal elimination unchanged	Generally avoid	ADVOR used a fixed 500 mg IV od dose and excluded eGFR < 20, so the reduced doses shown for lower eGFR strata are pragmatic extrapolations rather than trial-specified. Metabolic acidosis is the intended pharmacodynamic effect and is compounded by uremic acidosis in advanced CKD.
Steroidal MRA	Yes; outcomes evidence (RALES, EMPHASIS-HF)	Yes; spironolactone 25 mg od; K ≤ 5.0 mmol/L; monitor q2–4 weeks	Avoid (below ESC 2021 threshold of eGFR ≥ 30 mL/min/1.73 m^2^ and K ≤ 5.0 mmol/L [35]); restrict to specialist supervision if essential	Avoid; finerenone is not initiated below eGFR 25 per label and at eGFR < 15 should be considered case-by-case in specialist supervision regardless of indication	Spironolactone accumulates renally. Eplerenone: same K+ risk at equivalent MR-blocking dose. Finerenone preferred in DKD; heart–kidney tissue distribution proposed as basis for potentially more favorable hyperkalemia tolerability (pharmacological hypothesis, not confirmed in direct comparative trials); monitoring required with all MRAs. Potassium canrenoate (IV) is the only parenteral MRA option; 100–200 mg IV od–bid in routine clinical use, with escalation to 300–400 mg/day in selected refractory cases under close biochemical surveillance; licensed in several European countries (not available in the US) [23].
SGLT2i	Full CV and renal benefit; neurohormonal modulation and modest natriuretic adjunct	Full CV and renal benefit preserved; initiate without dose adjustment	Glycosuric/osmotic effect attenuated; neurohormonal and CV benefit may persist; empagliflozin label permits use to eGFR ≥ 20; dapagliflozin label ≥ 25 mL/min/1.73 m^2^	Glycosuric/osmotic component minimal; cardiovascular and neurohormonal benefits plausibly preserved (mechanistic inference, no outcome trials); off-label below current trial-inclusion thresholds (empagliflozin ≥ 20, dapagliflozin ≥ 25 mL/min/1.73 m^2^) and not routinely initiated	Not equivalent to thiazides as acute diuretic. Safe to initiate during hospitalization (meta-analysis OR 0.71 for 90-day all-cause death [48]).
Amiloride	Yes; 5–10 mg PO od	Yes; 5 mg od; monitor K; consider 2.5 mg below eGFR 45	2.5–5 mg; t½ markedly prolonged; K monitoring q2–3 days	Generally avoid; accumulation risk	Preferred over triamterene in Mg-depleted patients (conserves Mg). Particularly useful in nephrotic syndrome.
**(d). Electrolyte Management During Active Sequential Nephron Blockade**					
**Electrolyte**	**Target**	**Action Threshold**	**Route and Rate**	**Monitor After**	**Mechanism and Clinical Note**
Potassium	≥3.5 mmol/L ≥4.0 if digoxin or sustained VT/VF	K < 3.5 mmol/L: start oral replacement; K < 3.2 mmol/L or <3.5 in patients on digoxin or with sustained VT/VF: start IV replacement without waiting for oral route; verify and correct Mg first (hypokalemia is refractory to K alone when Mg is low)	10–20 mmol/h peripheral (max 40 mmol/L, e.g., 20 mmol in 500 mL); up to 20 mmol/h central with ECG monitoring (up to 40 mmol/h reported in ICU/critical-care settings with continuous monitoring)	Recheck 2–4 h after infusion; verify Mg ≥ 0.8 first	ROMK-mediated tubular K secretion requires intracellular Mg. Hypokalemia refractory to K replacement without prior Mg correction. Loop diuretics: ↑ distal Na delivery to ENaC → ↑ K secretion. Thiazides amplify this.
Magnesium	≥0.8 mmol/L throughout active diuresis	<0.8 mmol/L: correct before or simultaneously with K+ < 0.6 mmol/L: urgent replacement	2–4 g MgSO_4_ IV over 4–6 h; repeat until target; recheck 4 h post-infusion	Daily during active combination diuresis	Loop diuretics abolish lumen-positive potential in TALH, impairing paracellular Mg reabsorption. Thiazides: intracellular Mg depletion of DCT cells. Amiloride conserves Mg; triamterene increases urinary Mg excretion.
Chloride	≥96 mmol/L (mechanistic target; not formally prospectively validated as endpoint)	Cl < 96 with alkalosis: correct via KCl if K is also low, via 0.9% NaCl if Na ≥ 135, or via acetazolamide if hypochloremic alkalosis predominates Cl < 90 with Na < 135: HSS or tolvaptan (not thiazide)	If K low: correct as KCl (addresses both); Iif Na < 135: HSS 100 mL 3% NaCl over 30–60 min with furosemide; if Na normal, K normal: 0.9% NaCl judiciously	Recheck 6–8 h after HSS	Cl depletion activates WNK1/WNK4 independently of RAAS, upregulating NCC and worsening diuretic resistance. Acetazolamide promotes HCO_3_ excretion preferentially, preserving Cl. SALT-HF post hoc supports Cl correction in the hypochloremic subgroup (see Section 6.6) [82].
HCO_3_/Acid-base	Correct hypochloremic alkalosis to HCO_3_ 22–28 mmol/L	HCO_3_ > 30 with Cl < 96: alkalosis from loop–thiazide combination; add acetazolamide HCO_3_ < 18 during acetazolamide: expected; do not stop unless pH falls below approximately 7.30 (pragmatic threshold) or concurrent respiratory failure	Acetazolamide 500 mg IV od can counteract the alkalosis; monitor ABG if HCO_3_ falls below 18	ABG or VBG 12 h after acetazolamide initiation; daily during 3-day course	Metabolic acidosis from acetazolamide is the intended pharmacodynamic effect (proximal HCO_3_ wasting). The mild compensatory ↑ pCO_2_ is physiologically appropriate and does not warrant cessation in the absence of respiratory failure.
Creatinine/renal function	10–20% rise: expected, acceptable >30%: evaluate carefully >50%: pause escalation	Rising Cr + rising Hct + stable/rising albumin: hemoconcentration; continue diuresis; rising Cr + falling UO + acidosis + flat Hct: probable ATN; reduce loop, consider UF	FeUrea < 35%: pre-renal physiology: urinalysis for granular casts: if positive, ATN more likely	Recheck Cr every 12–24 h during active combination therapy	Ahmad et al. (Circulation 2018) [83,84]: worsening kidney function during aggressive diuresis is not associated with tubular injury and does not consistently predict adverse outcomes. Treat symptoms and volume status, not serum creatinine. Separately, eGFR dips with GDMT initiation are typically hemodynamic and do not require discontinuation [85].

(a): Natriuresis-guided decision algorithm for intravenous loop diuretic therapy in acute decompensated heart failure. Monitoring parameters, adequacy thresholds, and escalation actions are derived from trial-level data and consensus recommendations. The 2 h urinary sodium threshold (<70 mmol/L) is the PUSH-AHF protocol trigger and the <50 mmol/L threshold for dose doubling is proposed in [3]; these are pragmatic triggers and not interchangeable definitions of treatment failure. The 6 h urine output target (>100–150 mL/h) reflects widely used clinical practice [21]. All thresholds should be interpreted in clinical context. AKI: acute kidney injury; ATN: acute tubular necrosis; BMP: basic metabolic panel; Cl: chloride; Cr: creatinine; Hct: hematocrit; HCO_3_: bicarbonate; IV: intravenous; K: potassium; Mg: magnesium; Na: sodium; SGLT2i: sodium–glucose cotransporter 2 inhibitor; UNa: urinary sodium concentration; UO: urine output; (b): Biochemical phenotyping framework for selecting adjunctive agents in loop-diuretic-resistant acute decompensated heart failure. Each phenotype is defined by a characteristic pattern of urinary and serum electrolyte findings, reflecting the predominant mechanism of resistance. Preferred adjuncts and dosing are derived from trial-level evidence where available; observational or expert-consensus data are indicated. Phenotypes are not mutually exclusive; combinations of mechanisms are common in advanced decompensation. ADHF: acute decompensated heart failure; ADH: antidiuretic hormone; BUN: blood urea nitrogen; CKD: chronic kidney disease; DCT: distal convoluted tubule; DKD: diabetic kidney disease; eGFR: estimated glomerular filtration rate; ENaC: epithelial sodium channel; FENa: fractional excretion of sodium; FE: furosemide equivalent; HCO_3_: bicarbonate; HFrEF: heart failure with reduced ejection fraction; HSS: hypertonic saline solution; IV: intravenous; K: potassium; MRA: mineralocorticoid receptor antagonist; NCC: Na-Cl cotransporter; PO: per os (oral); RAAS: renin–angiotensin–aldosterone system; RCT: randomized controlled trial; SNB: sequential nephron blockade; UNa: urinary sodium concentration; UO: urine output; WNK: with-no-lysine kinase. (c): Diuretic agent selection and dose adjustment stratified by estimated glomerular filtration rate in acute decompensated heart failure. Recommendations reflect pharmacokinetic profiles, available efficacy evidence, and clinical guideline thresholds for each eGFR stratum. The ×2.5 intravenous multiplier applied to the outpatient oral dose in patients with a low eGFR or an elevated urine albumin-to-creatinine ratio is derived from the high-dose arm of the DOSE trial [75]; this ratio should be considered a minimum starting point rather than a ceiling. Recommendations for lower eGFR strata (15–29 and <15 mL/min/1.73 m^2^) are based on observational data and pharmacokinetic extrapolation where prospective trial data are unavailable. bid: twice daily; CKD: chronic kidney disease; CV: cardiovascular; DCT: distal convoluted tubule; eGFR: estimated glomerular filtration rate; HF: heart failure; HCTZ: hydrochlorothiazide; IV: intravenous; K: potassium; MRA: mineralocorticoid receptor antagonist; NCC: Na-Cl cotransporter; od: once daily; OAT: organic anion transporter; PO: per os (oral); RBC: red blood cell; SGLT2i: sodium–glucose cotransporter 2 inhibitor; SNB: sequential nephron blockade; t½: half-life; UACR: urine albumin-to-creatinine ratio. (d): Electrolyte monitoring targets and correction protocols during active sequential nephron blockade. Thresholds reflect physiological rationale rather than prospectively validated clinical endpoints, with the exception of potassium targets in patients receiving digoxin or with ventricular arrhythmia history. The chloride target (≥96 mmol/L) is a mechanistic threshold based on WNK kinase chloride-sensing biology; it has not been validated as a primary endpoint in a prospective interventional trial. Creatinine rise during active decongestion requires contextual interpretation: hemoconcentration (rising hematocrit and albumin) indicates effective volume removal and does not warrant diuretic reduction. ABG: arterial blood gas; ATN: acute tubular necrosis; Cl: chloride; Cr: creatinine; DCT: distal convoluted tubule; ECG: electrocardiogram; eGFR: estimated glomerular filtration rate; ENaC: epithelial sodium channel; FeUrea: fractional excretion of urea; HCO_3_: bicarbonate; Hct: hematocrit; IV: intravenous; K: potassium; Mg: magnesium; MgSO_4_: magnesium sulfate; Na: sodium; NCC: Na-Cl cotransporter; od: once daily; ROMK: renal outer medullary K+ channel; TALH: thick ascending limb of Henle; UO: urine output; VBG: venous blood gas; VF: ventricular fibrillation; VT: ventricular tachycardia; WNK: with-no-lysine kinase. ↑, increased.

**Table 4:** Personalized phenotypes of diuretic resistance and preferred sequential nephron blockade strategy.

Phenotype	Clinical/Biochemical Markers	Dominant Mechanism	Preferred Adjunct or Strategy	Agents to Avoid or Use with Caution	Evidence Level	Special Considerations in Older Adults/CKD
Hypochloremic metabolic alkalosis	Low chloride; high bicarbonate; prior high-dose loop or thiazide exposure	Chloride depletion with WNK activation and enhanced proximal sodium reabsorption	Acetazolamide	Advanced CKD; pre-existing acidosis; avoid below eGFR ≈ 20 mL/min/1.73 m^2^	Randomized (ADVOR) for decongestion; mechanistic for chloride repletion	Acidosis risk rises in advanced CKD and is unstudied below eGFR ≈ 20; ADVOR used a fixed 500 mg dose, so the reduction to 250 mg IV once daily at an eGFR < 40 is a pragmatic adjustment rather than a trial-specified one.
Distal nephron braking	Chronic loop exposure; poor urinary sodium after an adequate loop dose; preserved or moderately reduced eGFR	WNK-SPAK-mediated NCC upregulation and distal convoluted tubule remodeling	Metolazone, hydrochlorothiazide, chlorothiazide, or chlorthalidone, according to setting	Hyponatremia, hypokalemia, and hypomagnesemia; avoid thiazide if Na < 135 mmol/L	Randomized (CLOROTIC) for hydrochlorothiazide; observational for metolazone and chlorthalidone	Hyponatremia risk high in older adults (CLOROTIC median age 83 years); metolazone and chlorthalidone retain activity at low eGFR.
Dilutional hyponatremia with congestion	Hypervolemic hyponatremia; low serum sodium; persistent congestion	Non-osmotic arginine vasopressin excess with impaired free-water excretion	Tolvaptan	Rapid sodium correction; hepatic disease; frail patients with poor oral intake; avoid concurrent thiazide and initial fluid restriction	Label indication for hypervolemic hyponatremia; randomized (EVEREST) neutral for survival	Osmotic demyelination risk with rapid correction; cap correction at ≤8 mmol/L per 24 h in higher-risk patients.
Venous congestion-dominant	High VExUS grade; renal venous Doppler abnormalities; poor diuretic efficiency	Elevated central and renal venous pressure reducing net filtration and diuretic efficiency	Intensify loop-based decongestion with ultrasound-guided titration; ultrafiltration as salvage only	Avoid over-decongestion; ultrafiltration carries the adverse profile seen in CARRESS-HF	Observational (VExUS prognostication); single-center randomized data; hypothesis-generating; randomized (CARRESS-HF) for ultrafiltration	Intrarenal venous Doppler is attenuated in advanced CKD; over-decongestion is poorly tolerated in small, stiff ventricles.
Advanced CKD or impaired tubular delivery	eGFR < 30 mL/min/1.73 m^2^; high loop requirement; uremic competition for organic anion transporters	Impaired OAT-mediated tubular secretion with reduced intraluminal drug delivery	Intravenous loop optimization with higher-threshold dosing; adjunct chosen for retained low-GFR activity	Acetazolamide best avoided below eGFR ≈ 20; potassium-sparing agents used cautiously with RAAS blockade; evidence gaps	Mechanistic and extrapolated; advanced CKD under-represented in randomized cohorts	The defining CKD phenotype; KDIGO consensus notes that the patients most in need are least represented in trials.
Geriatric vulnerability	Frailty; orthostatic hypotension; falls; cognitive impairment; low albumin; polypharmacy	Reduced physiological reserve with altered volume and thirst regulation and heightened pharmacodynamic sensitivity	Lowest effective adjunct dose; closer monitoring; early de-escalation; functional endpoints	Aggressive diuresis; layered vasodilators; additive potassium-sparing and RAAS combinations	Expert consensus and observational	Delirium, falls, and volume depletion dominate; reconcile medications at discharge and review within 7–14 days (weight, BP, creatinine, Na, K, Mg, Cl).

Synthesis of the personalized, phenotype-driven framework for sequential nephron blockade in acute cardiorenal syndrome. Phenotypes are not mutually exclusive and frequently coexist in advanced decompensation, and the geriatric vulnerability phenotype may modify any of the others. The evidence level reflects the strongest source supporting each strategy within this review rather than a formal grading system, and detailed dosing and thresholds are provided in Table 3. Table 3b and Table 4 are complementary rather than duplicative. Table 3b is the operational tool and gives the preferred adjunct, its dose and its timing for each biochemical pattern, whereas Table 4 places the same phenotypes alongside the dominant mechanism, the agents to avoid, the level of supporting evidence and the modifications required in older adults and in chronic kidney disease. Abbreviations; BP, blood pressure; CKD, chronic kidney disease; eGFR, estimated glomerular filtration rate; NCC, sodium-chloride cotransporter; OAT, organic anion transporter; RAAS, renin–angiotensin–aldosterone system; SPAK, STE20/SPS1-related proline-alanine-rich kinase; VExUS, venous excess ultrasound; WNK, with-no-lysine kinase.

## Data Availability

No new data were created or analyzed in this study. Data sharing is not applicable to this article, as this is a narrative review based entirely on previously published sources, all of which are cited in the References section.

## Abbreviations

The following abbreviations are used in this manuscript:

ABG	arterial blood gas
ACEi	angiotensin-converting enzyme inhibitor
ADH	antidiuretic hormone
ADHF	acute decompensated heart failure
AHF	acute heart failure
AKI	acute kidney injury
AQ2	aquaporin-2
ARNI	angiotensin receptor–neprilysin inhibitor
ATN	acute tubular necrosis
AVP	arginine vasopressin
bid	twice daily
BMP	basic metabolic panel
BUN	blood urea nitrogen
CAI	carbonic anhydrase inhibitor
CCD	cortical collecting duct
CI	confidence interval
CKD	chronic kidney disease
Cl	chloride
Cr	creatinine
CV	cardiovascular
CYP2C9	cytochrome P450 2C9
CYP3A4	cytochrome P450 3A4
DCT	distal convoluted tubule
DKD	diabetic kidney disease
ECG	electrocardiogram
eGFR	estimated glomerular filtration rate
EMA	European Medicines Agency
ENaC	epithelial sodium channel
ESC	European Society of Cardiology
FDA	US Food and Drug Administration
FE	furosemide equivalent
FENa	fractional excretion of sodium
FeUrea	fractional excretion of urea
GDMT	guideline-directed medical therapy
GFR	glomerular filtration rate
HCO_3_	bicarbonate
Hct	hematocrit
HCTZ	hydrochlorothiazide
HF	heart failure
HFmrEF	heart failure with mildly reduced ejection fraction
HFpEF	heart failure with preserved ejection fraction
HFrEF	heart failure with reduced ejection fraction
HR	hazard ratio
HSS	hypertonic saline solution
INR	international normalized ratio
IV	intravenous
K	potassium
KCCQ	Kansas City Cardiomyopathy Questionnaire
KDIGO	Kidney Disease: Improving Global Outcomes
LOS	length of stay
LVEF	left ventricular ejection fraction
Mg	magnesium
MR	mineralocorticoid receptor
MRA	mineralocorticoid receptor antagonist
NA	not applicable
Na	sodium
NCC	Na-Cl cotransporter
NHE3	sodium–hydrogen exchanger isoform 3
NKCC2	Na-K-2Cl cotransporter 2
NP	natriuretic peptides
NS	not significant
NT-proBNP	N-terminal pro-B-type natriuretic peptide
OAT	organic anion transporter
od	once daily
OR	odds ratio
PCT	proximal convoluted tubule
PK	pharmacokinetics
PO	per os (oral)
PT	proximal tubule
RAAS	renin–angiotensin–aldosterone system
RBC	red blood cell
RCT	randomized controlled trial
ROMK	renal outer medullary K+ channel
ROS	reactive oxygen species
RR	relative risk
SGLT1	sodium–glucose cotransporter 1
SGLT2	sodium–glucose cotransporter 2
SGLT2i	sodium–glucose cotransporter 2 inhibitor
SNB	sequential nephron blockade
T2DM	type 2 diabetes mellitus
TALH	thick ascending limb of Henle
TGF	tubuloglomerular feedback
t½	half-life
UACR	urine albumin-to-creatinine ratio
UF	ultrafiltration
UGT	UDP-glucuronosyltransferase
UNa	urinary sodium concentration
UO	urine output
V2	vasopressin V2 receptor
VBG	venous blood gas
Vd	volume of distribution
VF	ventricular fibrillation
VT	ventricular tachycardia
WNK	with-no-lysine kinase
WRF	worsening renal function
